# Targeted genome editing of the non-model cyanobacterium *Cyanothece* PCC 7425 via CRISPR/Cas12a

**DOI:** 10.1007/s00253-026-13959-y

**Published:** 2026-07-11

**Authors:** Monis Athar Khan, Anne Durand, Fériel Skouri-Panet, Karim Benzerara, Corinne Cassier-Chauvat, Franck Chauvat, Soufian Ouchane

**Affiliations:** 1 Institute for Integrative Biology of the Cell (I2BC), Université Paris-Saclay, CEA, CNRS, Gif-Sur-Yvette, France; 2 UMR CNRS 7590. Institut de Minéralogie, de Physique Des Matériaux Et de Cosmochimie (IMPMC), Sorbonne Université, Muséum National d’Histoire Naturelle, Paris, France

**Keywords:** Non-model cyanobacteria, *Cyanothece*, CRISPR/Cas12a, Gene knockout

## Abstract

**Abstract:**

Cyanobacteria are diverse photosynthetic microorganisms of great interest for fundamental science and sustainable biotechnological applications. However, their polyploidy makes genetic manipulation challenging and time-consuming. The development of CRISPR/Cas tools has greatly accelerated genome editing and metabolic engineering of some cyanobacterial model species. In this work, we extend the CRISPR/Cas12a system for targeted gene deletion in the non-model cyanobacterium *Cyanothece* sp. PCC 7425, interesting for its ability to perform intracellular calcium carbonate (CaCO_3_) biomineralization, nitrogen fixation, etc. We demonstrate for the first time its tractability to gene knockout by generating deletion mutants of four genes (*cax3-cax4*, *gor*, and *sodB*) acting in metabolism and/or response to stresses, using Cas12a-mediated homologous recombination. Importantly, full chromosome segregation was rapidly achieved after a single round of selection in all cases. All mutants were genotypically and phenotypically characterised. Moreover, biochemical analysis in the case of the *ΔsodB* mutant further confirmed its targeted deletion. Overall, CRISPR/Cas12a provides a rapid and efficient system for genome editing in *Cyanothece* sp. PCC 7425, establishing this organism as a versatile model for studying oxidative stress pathways, metal toxicity, and moreover, the still poorly known mechanism(s) of intracellular CaCO_3_ biomineralization.

**Key points:**

• *Rapid and efficient CRISPR/Cas12a editing established in Cyanothece sp. PCC 7425.*

• *Fully segregated knockout mutants obtained after a single selection round.*

• *Platform for exploring the biotechnological potential of Cyanothece sp. PCC 7425.*

**Supplementary Information:**

The online version contains supplementary material available at https://doi.org/10.1007/s00253-026-13959-y.

## Introduction

Cyanobacteria have been pivotal in shaping Earth’s history and advancing our understanding of fundamental cellular processes. In comparison to algae and plants, cyanobacteria offer rapid growth and easier genetic manipulation, making them important model organisms for photosynthetic research. The ability of cyanobacteria to utilize water, CO₂, and sunlight as inputs to drive their metabolism positions them as an attractive chassis for “green and sustainable” biotechnological applications like bioremediation and bioproduction. Their remarkable diversity offers a variety of fascinating features, including adaptation to diverse habitats from marine and freshwater systems to extreme environments, different morphologies, wide variation in genome size, and considerable metabolic versatility, such as far-red light photo-acclimatation, mixotrophy, and nitrogen fixation (Cassier-Chauvat et al. 2021). Yet, research on cyanobacteria has largely focused on a few genetically tractable model species, including the unicellular cyanobacteria *Synechocystis* sp. PCC 6803 (hereafter, *Synechocystis* 6803), *Synechococcus elongatus* PCC 7942, *Synechococcus* sp. PCC 7002, and the filamentous cyanobacterium *Anabaena* sp. PCC 7120 (reclassified as *Nostoc* sp. PCC 7120) (Elhai and Wolk 1988; Golden et al. 1987; Kuhlemeier and van Arkel 1987; Marraccini et al. 1993). Despite considerable advances in the genetic toolbox available for model cyanobacterial species, efficient genome editing methods are still needed for non-model cyanobacteria, which often remain challenging to manipulate genetically.

The advent of CRISPR/Cas tools has greatly accelerating the genetic engineering of model cyanobacteria (Behler et al. 2018). Cas12a (previously Cpf1) from *Francisella novicida* was shown to be far less toxic than Cas9, leading to its development for genetic engineering of cyanobacteria (Ungerer and Pakrasi 2016; Wendt et al. 2016). Cas12a has additional advantages as compared to Cas9: (i) It creates a staggered cut with 5′ overhang as opposed to a blunt end generated by Cas9, promoting homology directed repair (Chauhan et al. 2023); (ii) Cas12a requires a shorter RNA as compared to dual crRNA-tracrRNA system used by Cas9 (Zetsche et al. 2015); and (iii) Cas12a recognizes a T-rich protospacer-adjacent motif (PAM), expanding its use in AT-rich genomes (Zetsche et al. 2015). The efficiency of the CRISPR/Cas12a system has been demonstrated by engineering marker-less knockouts, knockins and point mutations in *Synechococcus elongatus* UTEX 2973, *Synechocystis* 6803, and *Anabaena* sp. PCC 7120 (Niu et al. 2019; Ungerer and Pakrasi 2016). Its utility has been successfully extended to two fast-growing cyanobacteria *Synechococcus* sp. PCC 11801 (Sengupta et al. 2020) and *Synechococcus* sp. PCC 11901 (Victoria et al. 2024). CRISPR/Cas12a based gene deletion was also established in the marine cyanobacterium, *Synechococcus* sp. WH7803 (Capovilla et al. 2023). More recently, two non-model cyanobacteria, the unicellular nitrogen-fixing species *Cyanothece* sp. ATCC 51142 and the filamentous *Leptolyngbya* sp. strain BL0902, have also been found to be amenable to genetic manipulation using CRISPR/Cas12a to generate single/double gene knockouts (Bandyopadhyay et al. 2024; Gao et al. 2024).

In this study, we extended the application of CRISPR/Cas12a-based gene deletion to the non-model unicellular cyanobacterium, *Cyanothece* sp. PCC 7425 (hereafter, *Cyanothece* 7425). *Cyanothece* 7425 is able to fix nitrogen and generate H_2_ (Bandyopadhyay et al. 2011). It can produce cyanobactins, a class of ribosomal-synthesized cyclic peptides with significant pharmacological potential due to their diverse bioactivities, including anticancer, antimicrobial, and antiviral activities (Houssen et al. 2012; Khan and Phan 2025). It also harbours a panoply of antioxidant enzymes like glutathione disulfide reductase (Gor) and two superoxide dismutase (SOD) to deal with oxidative stress. Another salient feature of *Cyanothece* 7425 is its capability to form intracellular amorphous calcium carbonate (iACC) inclusions enclosed in a microcompartment (Benzerara et al. 2022, 2014). This process contributes to active sequestration of alkaline earth elements like calcium, strontium, barium, and radium, thus playing an important role in their biogeochemical cycling and possible implications for nuclear waste bioremediation (Blondeau et al. 2018; Mehta et al. 2019; Pamart et al. 2025). Several other cyanobacteria from diverse taxa have also been shown to form iACC inclusions (Benzerara et al. 2022, 2014). Moreover, the precipitation of calcium carbonate (CaCO_3_) by cyanobacteria also offers the potential for carbon capture and sequestration to reduce CO_2_ emissions (Jansson and Northen 2010). However, the lack of genetic tools in iACC-forming cyanobacteria has limited progress in elucidating the mechanisms underlying iACC formation.

Building on previous reports of RSF1010-derived plasmid transfer for gene overexpression (Chenebault et al. 2020), in this work we used another RSF1010-derived plasmid, pSL2680 (Ungerer and Pakrasi 2016), to develop targeted gene deletion in *Cyanothece* 7425 via CRISPR/Cas12a. We successfully deleted genes (*cax3-4*, *gor* and *sodB*) of varying length, including a ~ 3.7 kb deletion spanning two adjacent genes.

## Materials and methods

### Strains and culture conditions

All strains of *Cyanothece* 7425 were cultured at 30 °C under continuous agitation at 140 rpm (Infors rotary shaker) and standard light conditions: cool white light (Sylvania Luxline Plus) at an intensity of ~ 32 µmol photons m^−2^ s^−1^. Cells were grown in Mineral Medium (MM) (Domain et al., 2004), a modified version of the standard BG-11 medium (Stanier et al., 1971), containing 1.89 mM of sodium carbonate (Na₂CO₃) as the carbon source. For growth on plates, MM was solidified with 1% Bacto Agar (Difco). *E. coli* strain NEB 10-beta (New England Biolabs) was used for cloning and propagation of plasmids. The helper strain *E. coli* CM404 (Marraccini et al. 1993) was used for triparental conjugation. *E. coli* strains were grown in liquid or solid LB medium at 30 °C (CM404) or 37 °C (NEB 10-beta). The concentration of antibiotics used for selection were ampicillin (Amp) 100 µg mL^−1^, kanamycin (Km) 50 µg mL^−1^, streptomycin (Sm) 25 µg mL^−1^, and spectinomycin (Sp) 75 µg mL^−1^ for *E. coli*, and Sm 5 µg mL^−1^, Sp 5 µg mL^−1^, and Km 25 µg mL^−1^ for *Cyanothece* 7425.

### Plasmid construction

All cloning steps were carried out in the *E. coli* strain NEB10-beta. The primers are listed in Table S2. All enzymes used were from New England Biolabs (NEB) except AarI (Thermo Fisher Scientific). The plasmids were sequenced using the Mix2Seq kit (Eurofins Genomics). All plasmids used are listed in Table S1.

### pSL2680-trCas12a vector construction

The vector pSL2680 (Addgene plasmid # 85581) was a gift from Himadri Pakrasi (Ungerer and Pakrasi 2016). The vector was digested with EcoRI and BspEI restriction enzymes followed by blunting using DNA Polymerase I, Large (Klenow) Fragment, and ligation using T4 DNA ligase (NEB). The consequent vector pSL2680-trCas12a (Fig. S1) carries a ~ 1.8 kb internal deletion within the ~ 3.9 kb coding sequence of the *cas12a* gene removed from the pSL2680 vector.

### Editing vector construction

For the construction of vector pSL-*Δcax3-4*, first the complementary phosphorylated oligos cr-cax3-4_Fw/Rv (Integrated DNA Technologies) corresponding to the designed crRNA were annealed by heating at 65 °C. The annealed oligos were ligated at the AarI-linearized pSL2680 vector, generating vector pSL-crRNA-cax3-4. After successful cloning of the spacer, the homology platforms of ~ 600 bp lying both upstream the start codon of *cax3* and downstream the stop codon of *cax4* genes were generated by overlapping PCR (Phusion polymerase, NEB) on genomic DNA of *Cyanothece* 7425 using oligos F1-cax_Fw/Rv and F3-cax_Fw/Rv (Table S2), respectively. The Sm^R^Sp^R^ cassette was obtained by PCR using oligos F2-cax_Fw/Rv (Table S2) on purified pC plasmid (Chenebault et al. 2020). The three fragments were cloned at the SalI site in pSL-crRNA-cax3-4 by HiFi DNA assembly (NEB), generating the final vector pSL-*Δcax3-4* (Fig. S3).

The vector pSL-*Δgor* (Fig. S4) was constructed in a similar way except the homology repair template (~ 500 bp homology platforms) was recovered from synthesized plasmid pT-*Δgor* (Twist Bioscience, Table S1) using oligos F-pT-Δgor_Fw/Rv (Table S2). The vector pSL-*ΔsodB* (Fig. S5) was constructed as described for pSL-*Δcax3-4*. The repair template contained homology arms of ~ 500 bp upstream of the *sodB* start codon and downstream of its stop codon, flanking a Sm^R^Sp^R^ marker using the primers listed in Table S2.

### Transfer of plasmids via triparental conjugation

The plasmids were transferred from *E. coli* to *Cyanothece* 7425 via triparental conjugation as described previously (Chenebault et al. 2020). Five mL cultures of *E. coli* CM404 and NEB 10-beta (harbouring the desired plasmid) were grown overnight as described above. The next day, the cultures were washed with LB twice and then resuspended to 1.3 × 10^9^ cells mL^−1^ final concentration (*E. coli* OD_600nm_ 1 = 2 × 10^9^ cells mL^−1^). *Cyanothece* 7425 was grown as described above until OD_750nm_ 0.6- 1 and washed twice with MM. Then, cells were resuspended to a final concentration of 1.25 × 10^7^ cells mL^−1^ (Cyanothece 7425 OD_750nm_ 1 = 2.5 × 10^7^ cells mL^−1^). Then, 100 µL of this suspension was mixed with 30 µL suspension of each *E. coli* CM404 and *E. coli* NEB 10-beta carrying the desired plasmid (final cell number ratio of *E. coli*: *Cyanothece 7425* = 3:1). For the negative control, 100 µL of *Cyanothece 7425* was mixed with 60 µL LB medium to monitor the frequency of spontaneous antibiotic-resistant mutants. Finally, 30 µL aliquots of these mixtures were spotted on non-selective MM agar plates and incubated at 30 °C for 72 h under standard light (~ 32 µmol photons m^−2^ s^−1^). Following incubation, each spot was collected in 100 µL MM and spread onto selective MM plates containing either Sm and Sp 5 µg mL^−1^ or Km 25 µg mL^−1^ and incubated for 2–3 weeks under standard light conditions.

### Construction of mutant strains

The editing vectors were transferred to *Cyanothece* 7425 via triparental conjugation as described above. After 2–3 weeks of growth, all antibiotic-resistant clones were patched onto selective medium containing 5 µg mL^−1^ of both Sm and Sp. Their genomes were analysed by PCR to confirm deletion of the studied genes and full segregation of their chromosomes. At least two clones were then grown in liquid MM medium containing Sm and Sp 5 µg mL^−1^ for another round of PCR verification and sequencing (Eurofins Genomics).

### SEM/EDXS analysis of wild-type and *Δcax3-4*

Scanning electron microscopy (SEM) was performed at the IMPMC microscopy platform (Sorbonne Université, France), as described previously (Benzerara et al. 2014). Briefly, the wild-type and *Δcax3-4* strains were cultured in the BG11 medium until exponential phase (OD_750nm_ ~ 0.9 to 1.2). Then, 0.5 mL of cell suspension was collected onto 0.22 µm-pore-size polycarbonate filters (Millipore) and rinsed three times with ultrapure water. The filters were air-dried at room temperature and mounted on aluminium stubs using double-sided carbon tape. A thin film of carbon was deposited on the filters using a Leica EM SCD500 evaporator to enhance surface electron conductivity. The SEM apparatus was a Zeiss™ Ultra 55 microscope equipped with a field emission gun. Images were captured in the backscattered electron (BSE) detection mode using an angle-selective backscatter (AsB) detector. The acquisition conditions included an electron accelerating voltage set to 15 kV, a working distance of 7.5 mm, and a 60 μm aperture. Chemical mapping was conducted using energy-dispersive X-ray spectrometry (EDXS). Data was processed with the ESPRIT software (Bruker). SEM-EDXS analysis was replicated twice with independent biological replicates and at least three different areas observed each time.

### Spot viability assay

For the spot viability assay, the wild-type and mutant strains were grown in liquid MM until exponential phase (OD_750nm_ ~ 0.8–1.2). The cultures were centrifuged at 4500 g for 10 min and washed twice with sterile ultrapure water. The dilution series began with an OD₇₅₀ of 0.1, followed by five rounds of five-fold dilution. A drop of 10 µL from each dilution was spotted onto either MM agar plates or MM agar plates supplemented with different final concentrations (250 µM, 2.5 mM, and 25 mM) of CaCl_2_ for *Δcax3-4* and CuCl_2_ (5 µM), CdCl_2_ (2.5 µM), and CoCl_2_ (5 µM) for *Δgor*. The plates were incubated for 14 days under standard light (~ 32 µmol photons m^−2^ s^−1^) at 30 °C and imaged after. This experiment was repeated twice using independent biological replicates.

### SOD in-gel activity assay on non-denaturing gel

50 mL cultures of wild type (WT) and *ΔsodB* strains were grown until late exponential phase (OD_750nm_ ~ 1.5–1.8). Cells were prepared following the previously described protocol (Ivleva and Golden 2007). Protein concentration was determined by Pierce™ BCA Protein Assay Kits (Thermo Scientific) using BSA (Thermo Scientific) as standard. Then, 30 µg of total protein extracts was separated on a 16% non-denaturing polyacrylamide gel and stained for SOD activity as described (Weydert and Cullen 2010). The gel was first incubated in 20 mL solution of 50 mM Tris–HCl (pH = 7.2) with 0.85% TEMED (Sigma-Aldrich) and 56 µM Riboflavin-5-Phosphate (Sigma-Aldrich) for 15 min in light at room temperature (RT), followed by the addition of 2 mg mL^−1^ Nitroblue Tetrazolium Chloride (Sigma-Aldrich) and 15 min incubation in the dark at RT. The gel was washed twice in ultrapure water and left at RT in a light box until SOD-positive staining appeared.

### Disc diffusion assay

Five hundred microliters (OD_750nm_ = 0.8) of WT or *ΔsodB* were mixed with 6 mL semi-solid MM-agar (50%) and uniformly spread on MM-solidified agar plates. 6 mm sterile discs were soaked with 5 μL of 5 mM menadione (Sigma-Aldrich) and placed in the middle of the plate. Plates were incubated at 30 °C under standard light conditions (~ 32 µmol photons m^−2^ s^−1^) for 7 days prior to photography. The experiment was repeated three times using independently grown cultures of WT and *ΔsodB*.

### Verification of absence of editing plasmid

To check if the mutant strains have lost the editing vector, the WT, mutant strains, and Km^R^ and Sm^R^Sp^R^ positive control strains were grown in liquid medium until reaching mid-exponential phase. 10 µL of each culture was patched on MM agar plates containing either Sm and Sp 5 µg mL^−1^ or Km 25 µg mL^−1^. The plates were then incubated for 10 days under standard light at 30 °C and imaged after. Cells collected from the plates were then used for PCR verification with primers targeting the editing vector (See Table S2).

## Results

### Toxicity of Cas12a in *Cyanothece* PCC 7425

**Fig. 1:**
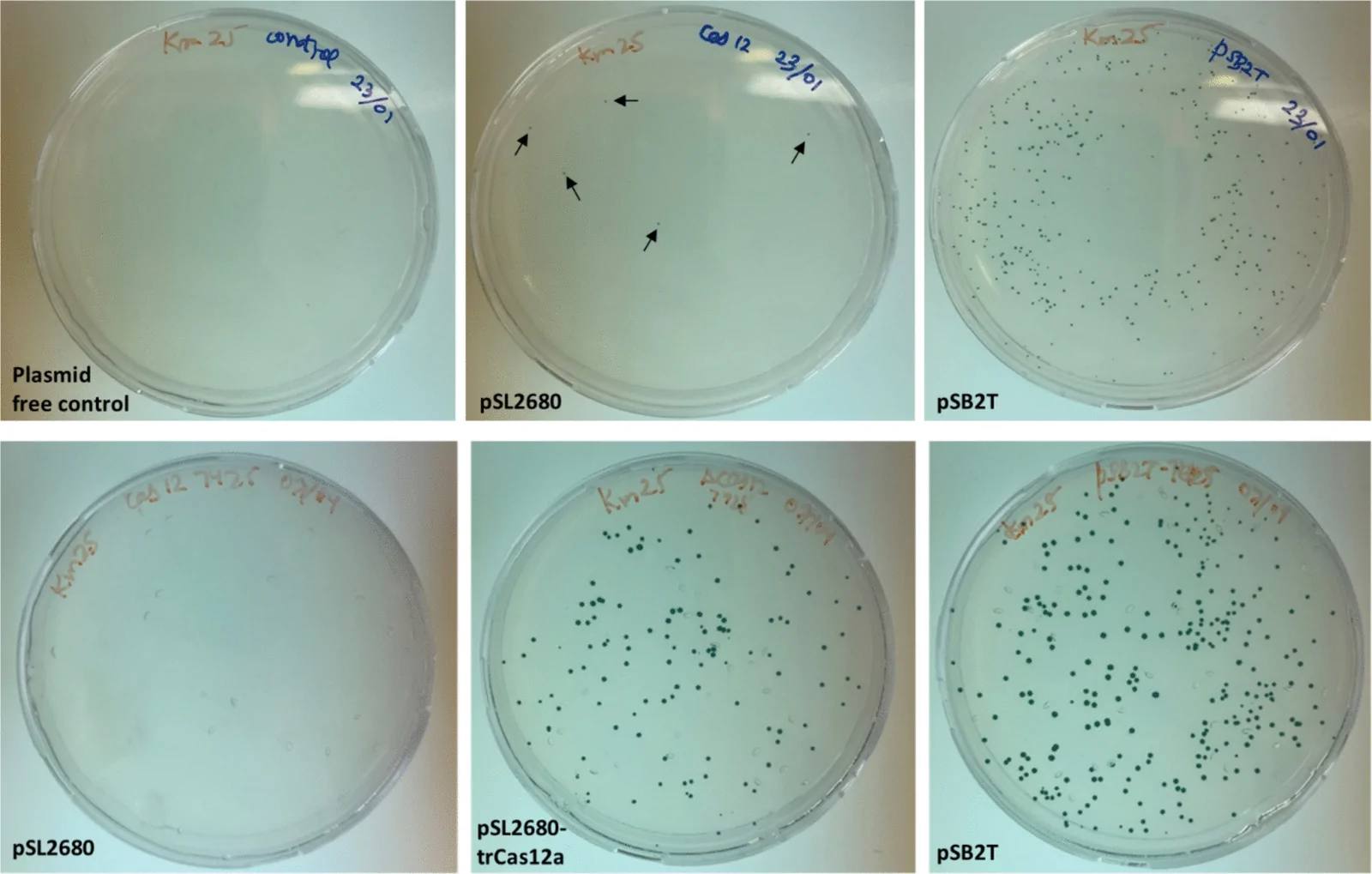
Conjugative transfer of plasmids into *Cyanothece* PCC 7425. Photographs of kanamycin-containing plates resulting from the conjugation with the plasmids pSB2T (positive control), pSL2680, pSL2680-trCas12 derivative constructed in this work, or no plasmid (negative control for checking possible Km^R^ spontaneous mutants). The Km^R^ clones obtained with the pSL2680 vector are indicated with arrows. The plates were imaged following 2 (upper row) or 3 (lower row) weeks of incubation

First, the potential toxicity of Cas12a expression in *Cyanothece* 7425 was assessed by introducing into it the pSL2680 vector (Ungerer and Pakrasi 2016) via triparental conjugation as we previously described (Chenebault et al. 2020). On several attempts, very few Km^R^ clones (2 to 6 clones) were obtained with pSL2680 as compared to the large number of clones (> 200) obtained with another RSF1010-derived replicative plasmid, pSB2T (Marraccini et al. 1993), used as a positive control (Fig. 1, top panel). The low number of clones obtained with pSL2680 suggested that the expression of Cas12a might be toxic to *Cyanothece* 7425. To test this hypothesis, the pSL2680-trCas12a plasmid encoding a truncated Cas12a protein was constructed. The trCas12a variant, missing the Cas12a amino acids 733–1330, lacks the RuvC and nuclease domains and hence cannot mediate DNA cleavage (Swarts 2019). Both pSL2680-trCas12a (Fig. S1) and pSL2680 were then conjugated into *Cyanothece* 7425. As expected, conjugation of the pSL2680-trCas12a vector produced much more clones (> 100), comparable to the positive control vector pSB2T (Fig. 1, bottom panel), suggesting potential Cas12a-associated toxicity. Nevertheless, the recovery of clones harbouring the pSL2680 plasmid (Fig. 1, top panel) indicated that the CRISPR/Cas12a system could be used to generate deletion mutants.

### CRISPR/Cas12a mediated deletion of antioxidant enzymes glutathione reductase and superoxide dismutase in *Cyanothece* PCC 7425: proof of concept

Cyanobacteria are frequently exposed to oxidative stress when performing oxygenic photosynthesis, which together with cellular respiration is a major source of reactive oxygen species (ROS) (Latifi et al. 2009). Under high light stress, an increased amount of ROS is produced, leading to inactivation of the photosynthetic machinery (Nishiyama et al. 2001). Among ROS, the superoxide anion (O₂•⁻) is detoxified through the action of superoxide dismutase (SOD) that transforms it into H_2_O_2_ and O_2_, and the H_2_O_2_ produced is further converted to H_2_O by catalases and peroxidases. Among the latter enzymes, glutathione peroxidases (Gpxs) reduce H_2_O_2_ to H_2_O using electrons provided by the antioxidant tripeptide glutathione (GSH) as a cofactor (Ursini et al. 1995). In this process, GSH is oxidized to glutathione disulfide (GSSG), which can be reduced to GSH by the NADPH-dependent enzyme glutathione reductase (Gor). Thus, Gor plays an important function in maintaining the cellular GSH pool. Overall, both Gor and SodB enzymes are critical for defence against oxidative stress in many organisms, including cyanobacteria (Cameron and Pakrasi 2010; Cassier-Chauvat et al. 2021; Latifi et al. 2009). To validate the potential use of CRISPR/Cas12a as a genetic tool to generate mutants in *Cyanothece* 7425, we used it to delete the *gor* and *sodB* genes.

**Fig. 2:**
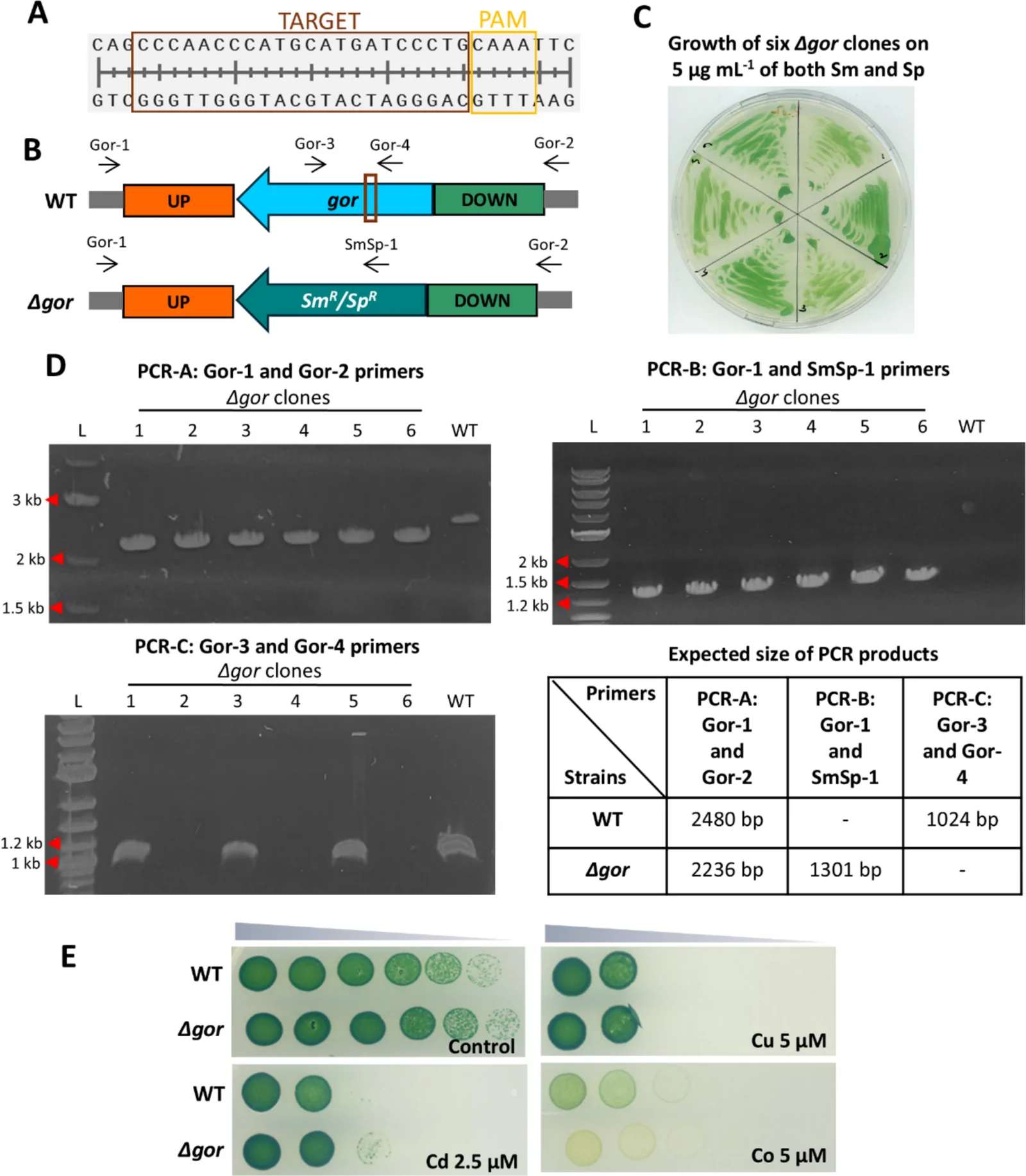
Targeted deletion of the *gor* gene in *Cyanothece* PCC 7425. **A** DNA sequence showing the crRNA target and the PAM site. **B** Schematic representation of the WT and *Δgor* chromosomes. The genes are represented by large arrows. The small black arrows represent the primers used for verification (Table S2), and the brown box indicates the target site of the crRNA. **C** Representative image of six *Δgor* clones grown for 10 days on plates containing Sm and Sp. **D** Three PCR strategies were employed to confirm successful deletion of the *gor* gene in six Sm^R^Sp^R^ clones. PCR products (expected sizes indicated in table) were resolved on 1% agarose gels. L denotes DNA ladder (1 kb plus, NEB). **E** Spot viability assay showing growth of the WT and *Δgor* strains on control (MM) medium and under metal stress conditions. The plates were incubated for 14 days under standard growth conditions and imaged after. This experiment was repeated twice using independent biological replicates

*Deletion of the gor gene*: Several previous attempts to delete the *gor* gene (KEGG ID: Cyan7425_3289) in *Cyanothece* 7425 using classical (CRISPR-independent) gene replacement via natural transformation did not yield any antibiotic-resistant colonies (unpublished data). Consequently, in this study we used the CRISPR/Cas12a system to delete the single *gor* gene in *Cyanothece* 7425. An important determinant of successful gene deletion using the CRISPR/Cas12a system is the sequence of the PAM site of the CRISPR target (Niu et al. 2019). These authors found that CRISPR targets with the PAM site 5′-TTTV-3′ (where V = A, G or C) exhibited a higher editing efficiency (95.2%) as compared to those with a 5′-GTTV-3′ PAM (75.9%). Therefore, for the deletion of *gor*, we selected the PAM site 5′-TTTG-3′ (Fig. 2A). For the recombination repair template, homology arms of ~ 500 bp upstream of the *gor* start codon and downstream of its stop codon, flanking a spectinomycin/streptomycin resistance (Sm^R^Sp^R^) marker were chosen (Fig. 2B). Hence, if the CRISPR/Cas12a system functions as intended, it would enable the full replacement of *gor* gene with the Sm^R^Sp^R^ cassette (Fig. 2B). The crRNA and the repair template were cloned into the pSL2680 vector to generate pSL-*Δgor*, (Fig. S4) which was subsequently introduced into *Cyanothece* 7425 via triparental conjugation. Selection was carried out on MM medium containing 5 µg mL^−1^ of both Sm and Sp. Upon conjugation of the editing plasmid pSL-*Δgor*, we obtained six clones that were patched onto selective medium containing 5 µg mL^−1^ of both Sm and Sp (Fig. 2C). PCR verification showed the clear presence of the mutant chromosome copies in all the clones and the correct integration of the Sm^R^Sp^R^ cassette in place of the target *gor* gene (Fig. 2D, PCR-A and B). However, PCR analysis with primers specific for the *gor* gene revealed that only three (clone n° 2, 4 and 6) of the six clones showed a complete absence of WT chromosome copies (Fig. 2D, PCR-C). The presence of WT chromosome copies in other clones (clone n° 1, 3 and 5) suggests that chromosome segregation has not yet been completed. For further phenotypic analysis, one fully segregated clone (clone n° 2) was chosen.

Under standard photoautotrophic conditions, the WT and *Δgor* mutant showed no growth differences (Fig. 2E). In cyanobacteria, the glutathione system has been shown to contribute to tolerance against metal stress (Cassier-Chauvat et al. 2023). Therefore, we compared the growth of WT and the *Δgor* mutant in the presence of toxic concentrations of cadmium (Cd), copper (Cu), and cobalt (Co) with a spot viability assay. As observed for the WT strain, the growth of the *Δgor* mutant was not impaired by both Cd and Cu (Fig. 2E). In contrast, while Co moderately affected the growth of the wild type, it induced a severe growth defect in the *Δgor* mutant (Fig. 2E).

**Fig. 3:**
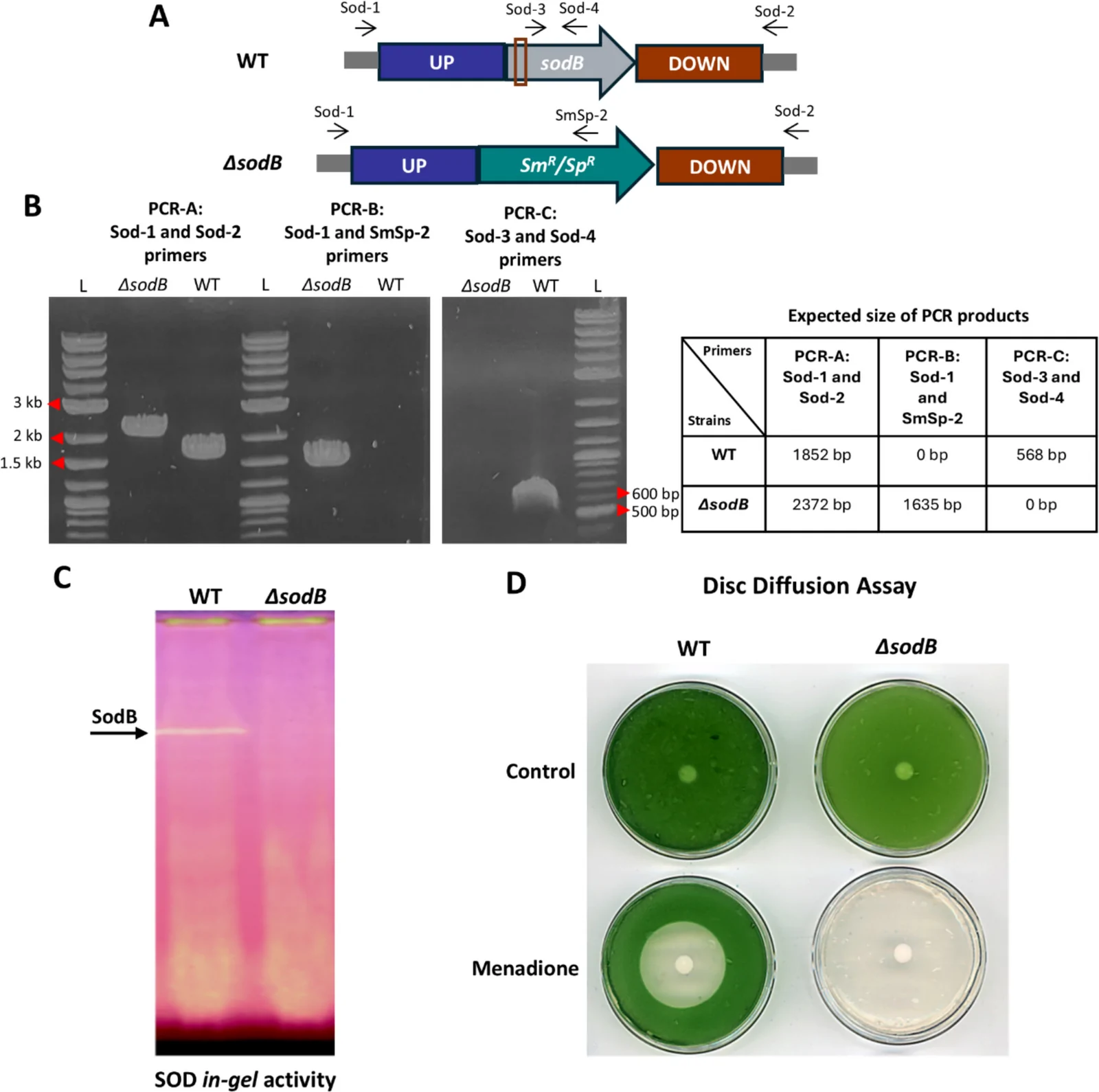
Targeted deletion of the *sodB* gene in *Cyanothece* PCC 7425. **A** Schematic representation of the wild-type (WT) and mutant (*ΔsodB*) chromosome. The genes are represented by large arrows pointing in the direction of their transcription. The small black arrows represent the primers used for verification (Table S2), and the brown box indicates the target site of the crRNA. **B** Three PCR strategies were employed to confirm successful deletion of the *sodB* gene. PCR products (expected sizes indicated in table) were resolved on 1% agarose gels. L denotes DNA ladder (1 kb plus, NEB). **C** The SOD *in-gel* activity of WT and *ΔsodB* was performed with 30 µg total protein extracts loaded on 16% native polyacrylamide gel. The band corresponding to SodB activity is indicated by the arrow.** D** Disc diffusion assay showing sensitivity of WT and *ΔsodB* strains to 5 mM menadione. The plates were incubated for 7 days under standard growth conditions and imaged after. The assay was repeated three times using independently grown cultures of the WT and *ΔsodB* strains

*Deletion of the sodB gen*e: Turning to the SOD family of antioxidant enzymes, it is known that depending on the metal co-factor present at their active site, SODs can be categorized into four different isoforms: MnSODs containing manganese, FeSODs containing iron, NiSODs containing nickel, and CuZnSODs containing copper/zinc (Boden et al. 2021). The different SODs also vary in terms of their subcellular localization. The genome of *Cyanothece* 7425 encodes two putative superoxide dismutases based on sequence homology: a membrane-associated MnSOD (*sodA*; KEGG ID: Cyan7425_3014) and a cytosolic FeSOD (*sodB*; KEGG ID: Cyan7425_1660). Given the central housekeeping role of *sodB* in the cytoplasm, where it is primarily responsible for scavenging superoxide radicals generated mainly at photosystem I and protecting cells from damage to 4Fe-4S cluster-containing proteins (Herbert et al. 1992; Keyer and Imlay 1996), we targeted the *sodB* gene in *Cyanothece* 7425 to further establish a CRISPR/Cas12a system for gene deletion in this species. The CRISPR target and the editing plasmid were designed as described above for the *gor* gene for the replacement of *sodB* with the Sm^R^Sp^R^ cassette (Fig. 3A). Following conjugation of the plasmid pSL-*ΔsodB* (Fig. S5), we obtained only a single clone, which was found to be fully segregated and the Sm^R^Sp^R^ cassette integrated in place of the *sodB* gene (Fig. 3B, PCR-A and B). Furthermore, PCR with primers specific for the *sodB* gene resulted in no amplification in the *ΔsodB* mutant (Fig. 3B, PCR-C). This suggests that SodB is dispensable for the growth of *Cyanothece* 7425.

A practical advantage of targeting the *sod* genes is that the SOD enzymatic activity can be readily detected using an *in-gel* colorimetric assay. *In-gel* SOD activity assay revealed a single band in the wild-type strain, which was completely absent in the *ΔsodB* mutant (Fig. 3C). Moreover, we assessed the susceptibility of the *ΔsodB* mutant to menadione using a disc diffusion assay. For the wild-type strain, a growth inhibition zone of 15.1 mm in diameter was observed in the presence of 5 mM menadione. In contrast, the growth of the *ΔsodB* mutant was completely abolished, indicating its hypersensitivity to menadione (Fig. 3D).

### Deletion of two Ca^2+^/H^+^antiporter encoding *cax* genes: are *cax *genes involved in iACC formation in *Cyanothece* PCC 7425?

Although the genetic determinants of iACC formation remain unknown, some proteins related to calcium transport have been identified (Bruley et al. 2025). Among calcium transporters, the Ca^2^⁺/H⁺ antiporter (Cax) appears to be an interesting candidate. In cyanobacteria, Cax proteins are usually involved in exporting Ca^2+^ from the cytoplasm to the periplasm, playing an important role in Ca^2+^ homeostasis (Waditee et al. 2004). Interestingly, the expression of one of the *cax* genes is directly involved in CaCO_3_ biomineralization in the coccolithophore microalga *Emiliania huxleyi*, where the encoded Cax protein is suggested to transport Ca^2+^ into the intracellular biomineralization compartment known as the coccolith vesicle (Mackinder et al. 2011). Therefore, we hypothesized that the Cax proteins in *Cyanothece* 7425 may similarly participate in Ca^2+^ trafficking within the bacterium, thereby contributing to iACC formation.

**Fig. 4:**
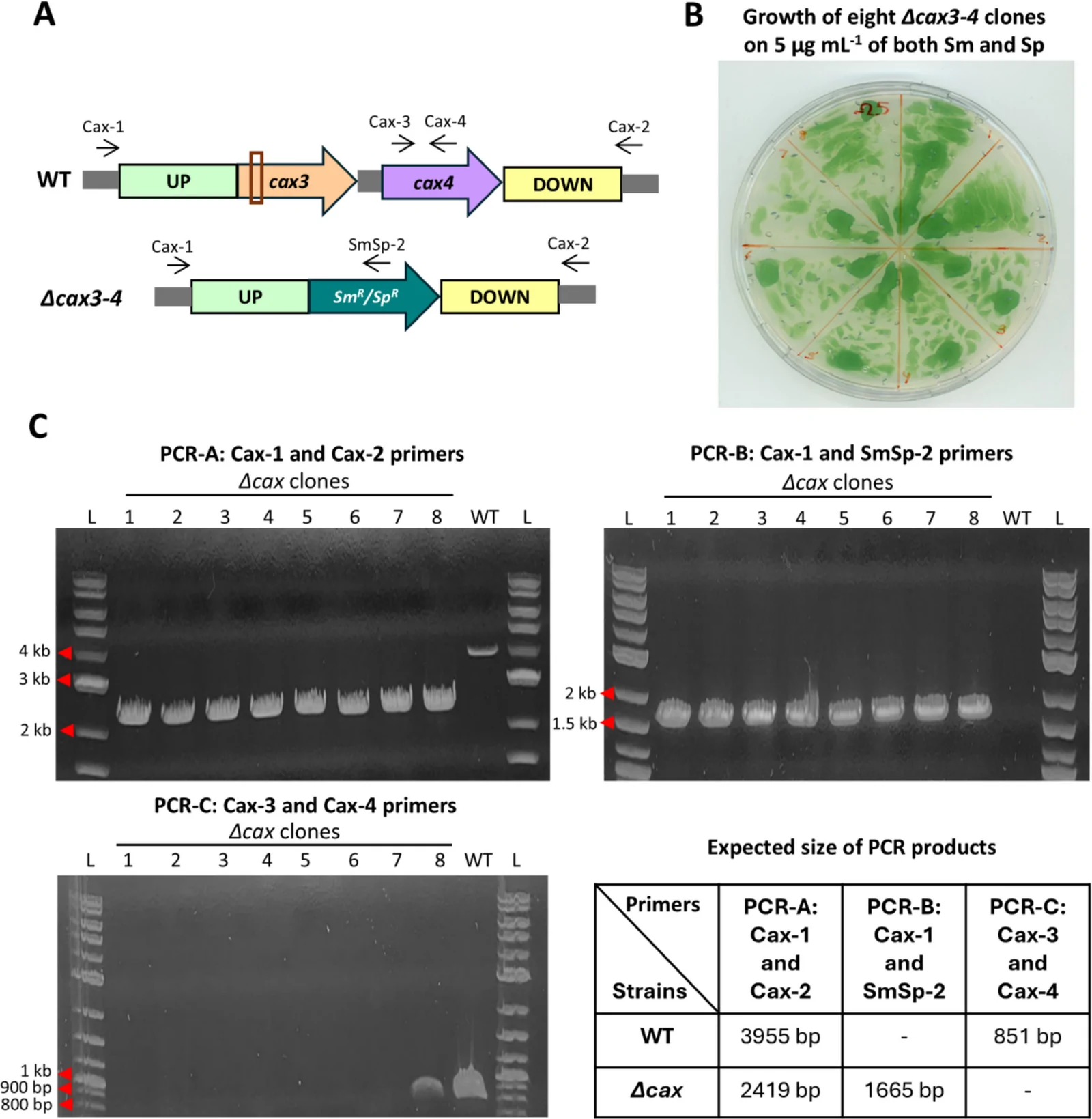
Targeted deletion of *cax3* and *cax4* genes in *Cyanothece* PCC 7425. **A** Schematic representation of the wild-type (WT) and mutant (*Δcax3-4*) chromosome. The genes are represented by large arrows pointing in the direction of their transcription. The small black arrows represent the primers used for verification (Table S2), and the brown box indicates the target site of the crRNA. **B** Representative image of eight *Δcax3-4* clones grown for 10 days in the presence of both Sm and Sp. The plates were imaged 10 days following incubation. **C** Three PCR strategies were employed to confirm successful deletion of the *cax3* and *cax4* genes in eight Sm^R^Sp.^R^ clones. PCR products (expected sizes indicated in table) were resolved on 1% agarose gels. L denotes DNA ladder (1 kb plus, NEB)

The genome of *Cyanothece* PCC 7425 contains four putative *cax* genes (KEGG ID: Cyan7425_0927, Cyan7425_1779, Cyan7425_3000, and Cyan7425_3001, named *cax1-4*, respectively). Amongst them, *cax3* (Cyan7425_3000) and *cax4* (Cyan7425_3001) are in a genomic region that also harbours other genes potentially involved in Ca^2+^ transport (Fig. S2). The adjacent *cax3* and *cax4* genes were chosen as primary targets, as their deletion was expected to perturb Ca^2+^ homeostasis and hence affect iACC formation. Successful recombination should result in the replacement of the *cax3-cax4* gene cluster by the Sm^R^Sp^R^ selection marker (Fig. 4A). The editing plasmid pSL-*Δcax3-4* (Fig. S3) was subsequently introduced into *Cyanothece* 7425 via triparental conjugation. After 3 weeks, eight colonies were obtained and subsequently patched onto selective medium. All colonies grew well under standard conditions (Fig. 4B) and were subjected to PCR verification to check for successful deletion of *cax3* and *cax4* genes. As shown in Fig. 4C, all tested clones harboured only mutant chromosome copies, with no WT chromosome detected, confirming *cax3-4* deletion (PCR-A) upon their replacement by the Sm^R^Sp^R^ cassette (PCR-B). To further confirm complete segregation of the chromosome, PCR with primers targeting the *cax4* gene confirmed that in seven out of eight clones, the *cax3-4* genes were fully deleted (Fig. 4C, PCR-C). Overall, these results indicate that the *cax3* and *cax4* genes are not vital for growth under standard conditions.

To assess the impact of *cax3* and *cax4* knockout on iACC formation, one fully segregated clone (clone n°1) was selected, and SEM-EDXS analysis was conducted. No obvious differences in iACC inclusions were observed between the WT and the *Δcax3-4* under the conditions tested (Fig. S6A), suggesting that the deletion of *cax3-4* genes does not markedly affect iACC formation. Next, to assess whether *cax3-4* might be involved in Ca^2+^ export from the cell, we performed a spot viability assay to compare the growth of WT and the *Δcax3-4* mutant under high calcium conditions. Even at 25 mM Ca^2+^ (about 100 times the normal concentration in standard MM medium), no significant differences in growth were observed between the WT and mutant strains (Fig. S6B).

### Verification of absence of the pSL2680-derived editing plasmids in the deletion mutants

**Fig. 5:**
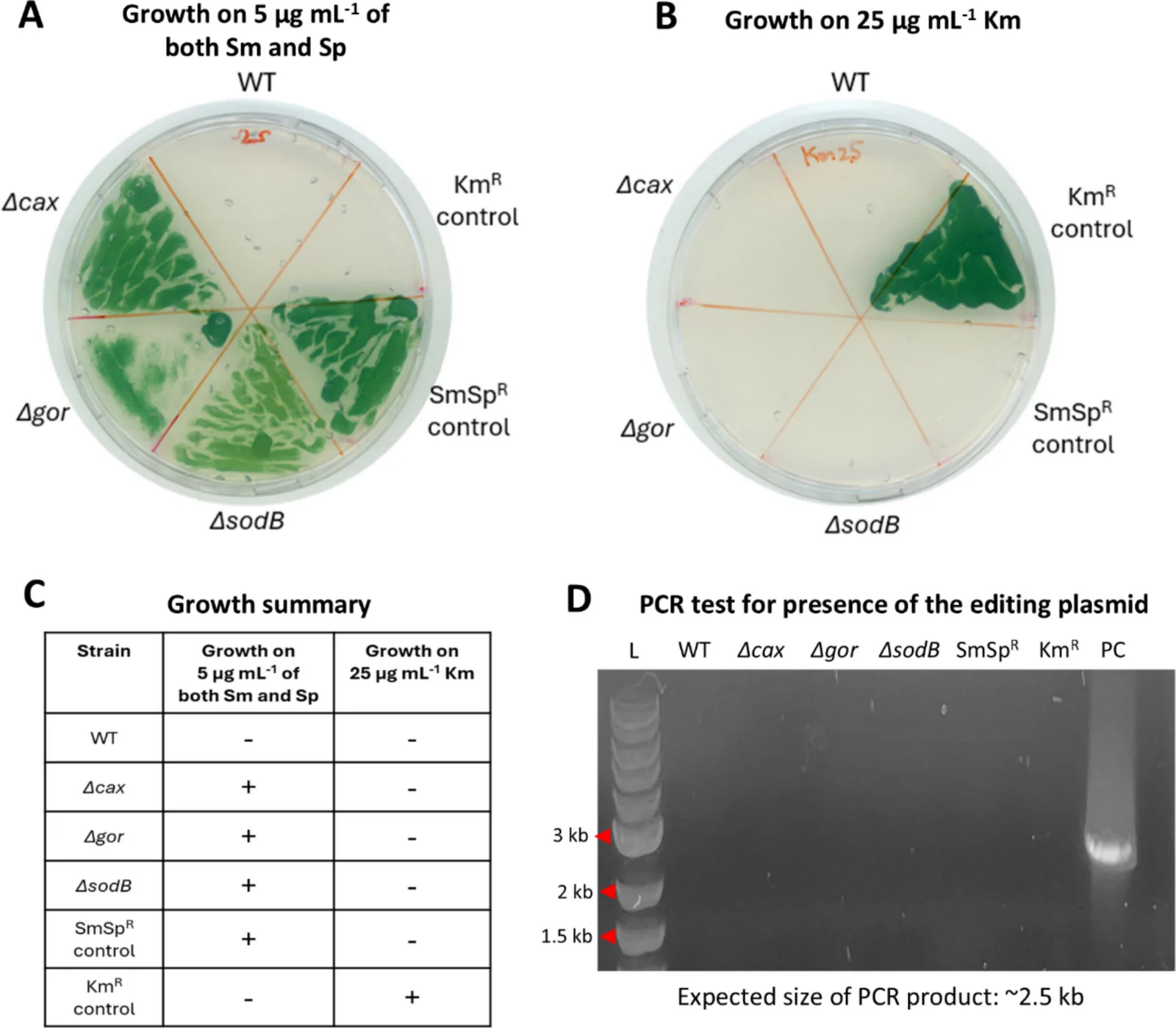
Verification of presence of the editing plasmid. The growth of the indicated strains was assessed on selective media containing either (**A**) streptomycin and spectinomycin or (**B**) kanamycin. **C** The table summarises whether each strain exhibited growth (+) or no growth (−) on the respective selective media. **D** PCR analysis using primers (Table S2) targeting the editing vector, showing the products resolved on 1% agarose gels. PC is positive control. L denotes DNA ladder (1 kb plus, NEB)

To determine whether the deletion mutants have been cured of their respective pSL2680-derived editing plasmid, these strains were patched onto selective media containing either streptomycin and spectinomycin (Fig. 5A) or kanamycin (Fig. 5B). Since the backbone of the editing pSL2680 plasmid carries a kanamycin resistance marker (Fig. S1), the loss of the plasmid results in an inability of the mutant strains to grow in presence of kanamycin. Consistently, all three mutants (*Δcax3-4, Δgor* and *ΔsodB*) failed to grow in the presence of kanamycin (Fig. 5B and C). In addition, PCR verification using primers specific for the editing plasmids was negative, confirming the loss of these editing plasmids (Fig. 5D).

## Discussion

The low number of colonies obtained with the pSL2680 vector suggests that strong Cas12a expression may influence the cell viability of *Cyanothece* 7425. In cyanobacteria, Cas9 toxicity has been proposed to result from off-target DNA cleavage that cannot be efficiently repaired, ultimately leading to cell death (Wendt et al. 2016). Although the underlying mechanism remains unclear, the potential Cas12a-associated toxicity observed in *Cyanothece* 7425 may arise from similar factors. Our finding is consistent with a few other studies that reported the toxicity associated with high expression of dCas12a (deactivated Cas12a that binds to DNA, hindering gene expression) (Knoot et al. 2020; Xie et al. 2025). Nevertheless, the use of inducible promoters to fine-tune Cas12a expression would enable a more precise assessment of its potential toxicity and could also help minimize any negative effects associated with high expression to improve overall gene-editing efficiency (Victoria et al. 2024).

Despite the observed toxicity, the CRISPR/Cas12a system is rapid and efficient for genome editing in *Cyanothece* 7425. The targeted genes can be replaced with an Sm^R^Sp^R^ cassette through homologous recombination following the double-stranded break generated by Cas12a at the desired genomic locus. While here we show that homology arms of approximately ~ 500 bp were sufficient for homologous recombination, previous studies have reported that longer flanking regions (> 750 bp) can further improve homologous recombination efficiency in cyanobacteria (Ruffing et al. 2016). Therefore, the use of extended homology arms represents a useful strategy for further optimizing genome editing efficiency in *Cyanothece* 7425.

More importantly, for all target genes (*cax3-4*, *gor* and *sodB*), full chromosome segregation was achieved after the first patch. While the copy number of *Cyanothece* 7425 remains unknown, some freshwater cyanobacteria are polyploid and contain multiple chromosome copies (Watanabe 2020), making segregation a time-consuming process that may require repeated rounds of subculturing and selection. However, CRISPR/Cas-based systems can help overcome this limitation by increasing editing efficiency through Cas-induced double-strand breaks that promote homology-directed repair, particularly in cyanobacteria where non-homologous end joining is absent in many species (Behler et al. 2018; Rajaram et al. 2020).

Plasmid curing is an essential step for repeated genome editing in cyanobacteria due to limited antibiotic markers and slow growth, which makes multiple subculturing in antibiotic-free media time-consuming (Dias et al. 2015). Here, we hypothesize that selecting solely for the Sm^R^/Sp^R^ cassette encoded in the repair template and not for the Km^R^ marker on the editing plasmid during the initial selection step, combined with the associated potential toxicity of Cas12a, enriched for cells that had undergone both successful recombination and loss of the plasmid. Curing of a Cas12a-based plasmid can be further accelerated through the use of *sacB*-mediated sucrose counter-selection as described in (Niu et al. 2019).

Most, if not all, freshwater cyanobacteria harbour a *sodB* gene that has been shown to be essential for cell growth of *Synechocystis,* which harbours no other SOD-encoding gene (Ke et al. 2014). Interestingly, we show that the *sodB* is not essential for the growth of *Cyanothece* 7425 under standard phototrophic conditions. These results suggest that superoxide radicals can be handled by not only SodB, but also very likely by other scavengers yet to be identified. However, the cell death of *ΔsodB* mutant in the presence of menadione clearly indicates that SodB plays an important role in the protection of *Cyanothece* 7425 against heightened oxidative stress. Similarly, the absence of Gor did not affect the growth of *Cyanothece* 7425, consistent with observations in both *E. coli* and yeast (Apontoweil and Berends 1975; Muller 1996). This suggests that either increased GSH synthesis or the thioredoxin reductase/thioredoxin system might compensate for the absence of GSSG reduction to GSH (Cameron and Pakrasi 2010; Cassier-Chauvat et al. 2023; Prinz et al. 1997). The sensitivity of *Cyanothece* 7425 to cobalt, exacerbated in the *Δgor* mutant, is consistent with that observed in *Synechocystis* PCC 6803, where the glutathione system has been demonstrated to cope with cobalt-induced toxicity (Marceau et al. 2024; Morette et al. 2026). Indeed, in *Synechocystis* PCC 6803, knockout of GSH-dependent GST (Glutathione S-transferase) resulted in increased sensitivity to Co, accompanied by impaired Fe homeostasis and disruption of 4Fe-4S clusters (Marceau et al. 2024). Further investigation is necessary to better understand by which mechanisms cyanobacteria tolerate Co and the specific role of Gor in mitigating Co-induced toxicity.

Under the conditions tested, the deletion of *cax3-cax4* did not result in obvious differences in iACC formation compared with the WT. However, further mechanistic studies are required to fully elucidate the contribution of these genes to iACC biomineralization. It is possible that the above-mentioned Cax1 and Cax2 proteins provide functional redundancy and compensated for the loss of both Cax3 and Cax4. Given the growth of iACC-forming cyanobacteria is highly Ca^2^⁺ dependent, Ca^2^⁺ trafficking is likely complex in these cyanobacteria (De Wever et al. 2019). Other transporters, such as Ca^2^⁺-associated P-type ATPases, can contribute to Ca^2^⁺ flux in *Cyanothece* 7425, and may modulate or mask the *Δcax3-4* mutant phenotype. Interestingly, a new gene family, *ccyA,* located in the same locus as *cax3-4* and adjacent to three putative P-type ATPases in *Cyanothece* 7425 (Fig. S2), has been identified as a marker of iACC formation, and may play a role in Ca^2^⁺ transport and/or formation of CaCO_3_ inclusions (Benzerara et al. 2022). In the iACC-forming cyanobacterium *Microcystis aeruginosa* PCC 7806, genes encoding Cax also co-localized and co-expressed with *ccyA* (Bruley et al. 2025). While these observations indicate a functional association between these factors, direct mechanistic evidence for their involvement in Ca^2^⁺ trafficking or iACC formation is currently lacking. CRISPR/Cas12a-mediated mutagenesis of these putative ATPase genes and *ccyA* is currently underway in our laboratory and is expected to clarify their roles in iACC formation in *Cyanothece* 7425.

In the present study, although previous attempts to generate mutants in *Cyanothece* 7425 using conventional homologous recombination were unsuccessful, CRISPR/Cas12a enabled the generation of fully segregated deletion mutants for all targeted genes in a single round of selection, highlighting its potential as an effective genome editing strategy for this non-model cyanobacterium. Given the diverse and physiologically relevant traits of *Cyanothece* 7425, including intracellular amorphous calcium carbonate biomineralization, metal tolerance, and nitrogen fixation, this work provides a foundation for the future development of CRISPR/Cas12a-based approaches to generate both marker-based and marker-less mutants as well as heterologous gene integration to enable functional studies and biotechnological exploitation of this cyanobacterium.

## Conclusion

The biodiversity of non-model microorganisms plays a crucial role in expanding our understanding of fundamental metabolism and uncovering novel pathways and metabolites. Many of these organisms possess unique metabolic traits, making them valuable resources for biotechnology and therapeutic applications. However, their effective study and exploitation depend on improving their cultivation under laboratory conditions and genetic tractability. In this context, we extended the use of a CRISPR/Cas12a-based system to achieve targeted gene knockouts in the non-model cyanobacterium *Cyanothece* 7425. This bacterium is notable because of its ability to form iACC inclusions, a process relevant to the biogeochemical cycling of alkaline earth elements. The establishment of these new genetic tools represents a significant step forward in elucidating the molecular mechanisms underlying iACC formation in cyanobacteria**.** Our ongoing studies on other genes possibly involved in intracellular biomineralization will further our understanding of this fascinating process.

## Supplementary Information

Below is the link to the electronic supplementary material.

ESM 1 (PDF 2.98 MB)

## Data Availability

No datasets were generated or analysed during the current study.
