# Supplementary material for: Targeted genome editing of the non-model cyanobacterium Cyanothece PCC 7425 via CRISPR/Cas12a
Source: Appl Microbiol Biotechnol. 2026 Jul 11;110:299. doi: 10.1007/s00253-026-13959-y (PMC13642857; doi:10.1007/s00253-026-13959-y)
Supplement: Supplementary file 1 [file 253_2026_13959_MOESM1_ESM.pdf]

Targeted genome editing of the non-model cyanobacterium *Cyanothece* PCC 7425 via CRISPR/Cas12a

KHAN Monis Athar[1*] (ORCID: 0009-0008-4161-5913), DURAND Anne[1] (ORCID: 0000-0002-0463-5930), SKOURI-PANET Feriel[2] (ORCID: 0000-0002-2623-9058), BENZERARA Karim[2] (ORCID: 0000-0002-0553-0137), CASSIER-CHAUVAT Corinne[1] (ORCID: 0000-0002-9151-7667), CHAUVAT Franck[1] (ORCID: 0000-0001-6901-5559), OUCHANE Soufian[1*] (ORCID: 0000-0002-8161-3866)

[1] Université Paris-Saclay, CEA, CNRS, Institute for Integrative Biology of the Cell (I2BC), Gif-sur-Yvette, France

[2] Sorbonne Université, Muséum National d'Histoire Naturelle, UMR CNRS 7590. Institut de Minéralogie, de Physique des Matériaux et de Cosmochimie (IMPMC), Paris, France

*Corresponding authors

Correspondence to: soufian.ouchane@i2bc.paris-saclay.fr / monis-athar.khan@i2bc.paris-saclay.fr

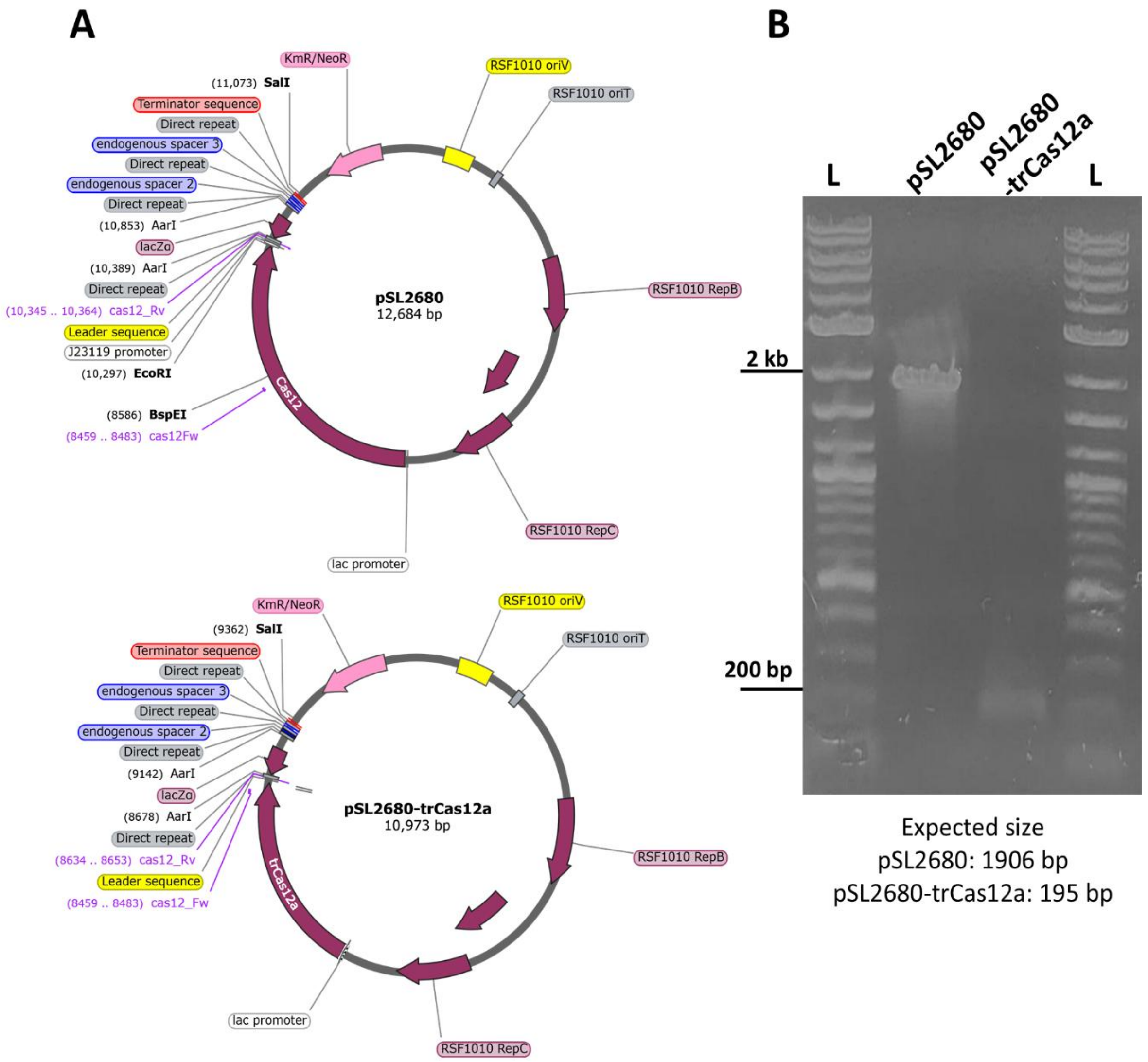

Figure S1: (A) The map of the pSL2680 (top panel) and pSL2680-trCas12a (bottom panel) plasmids, made using SnapGene. **(B)** PCR amplification of pSL2680 and pSL2680-trCas12a with Cas12-1 and Cas12-2 primers (Table S2) resolved on a 1% agarose gel indicating the corresponding product sizes. L denotes DNA ladder (1 kb plus, NEB).

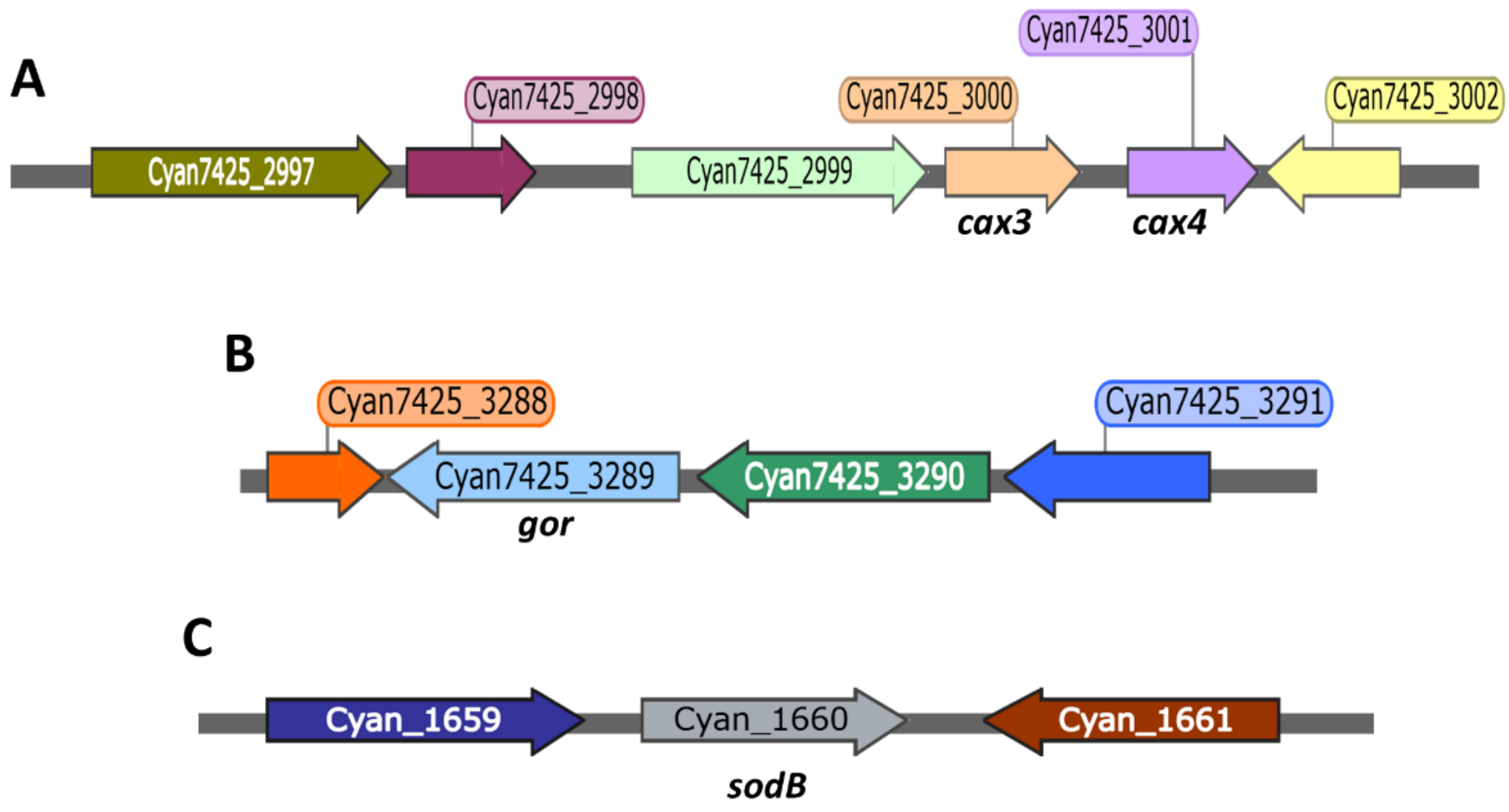

Figure S2: (A) Genomic region around the *cax3* (Cyan7425_3000) and *cax4* **(**Cyan7425_3001) genes. The upstream genes Cyan7425_2999 Cyan7425_2998 and Cyan7425_2997 encode a putative P-type ATPAse, protein of unknown function, and another putative P-type ATPAse, respectively. The downstream gene Cyan7425_3002 encodes an anion transporting ATPase. **(B)** Genomic region around the *gor* gene **(**Cyan7425_3289) showing its upstream gene Cyan7425_3288 (hypothetical protein and its downstream genes Cyan7425_3290 (nucleotide sugar dehydrogenase), Cyan7425_3291 (NAD-dependent epimerase). **(C)** Genomic region around the *sodB* gene (Cyan7425_1660) showing its flanking genes Cyan7425_1659 (hypothetical protein) and Cyan7425_1661 (dihydroorotase).

The genes are represented by large arrows pointing in their direction of transcription. The maps were made using SnapGene.

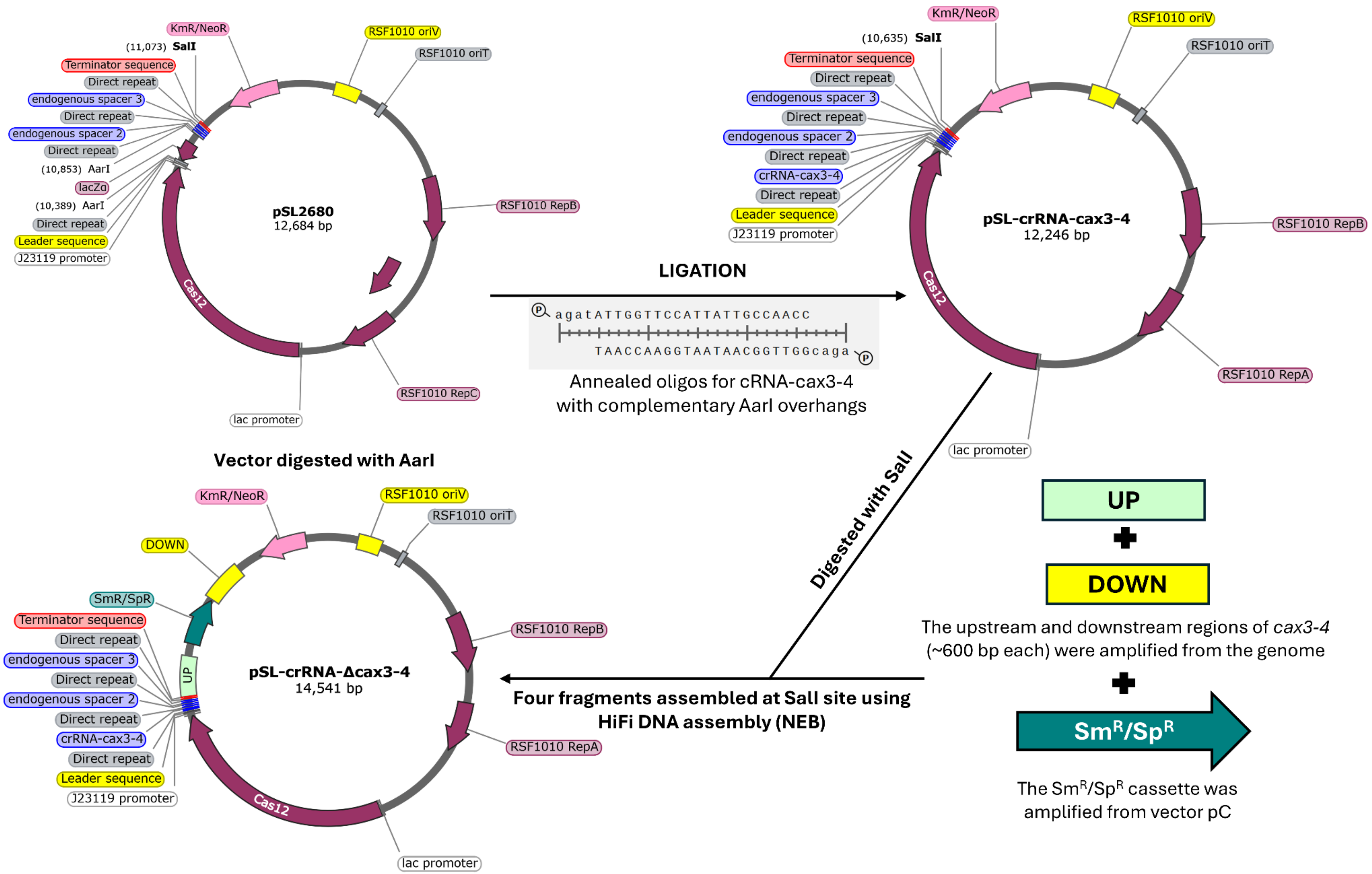

Figure S3: Schematic representation of the construction of pSL-*Δcax3-4* editing plasmid. The primers used are listed in Table S2. The pSL-*ΔsodB* editing plasmid was constructed similarly.

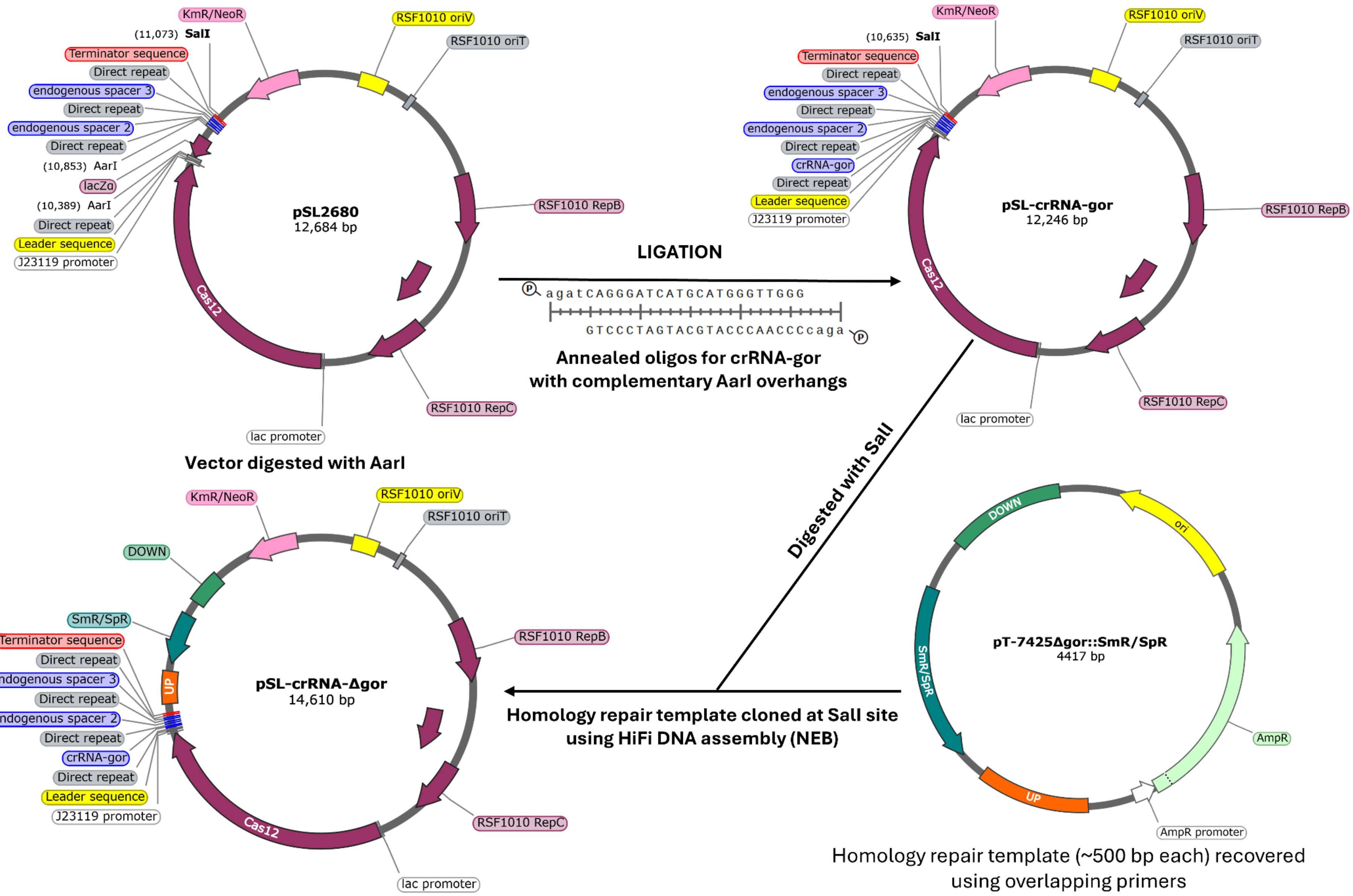

Figure S4: Schematic representation of the construction of pSL-*Δgor* editing vector. The primers used are listed in Table S2.

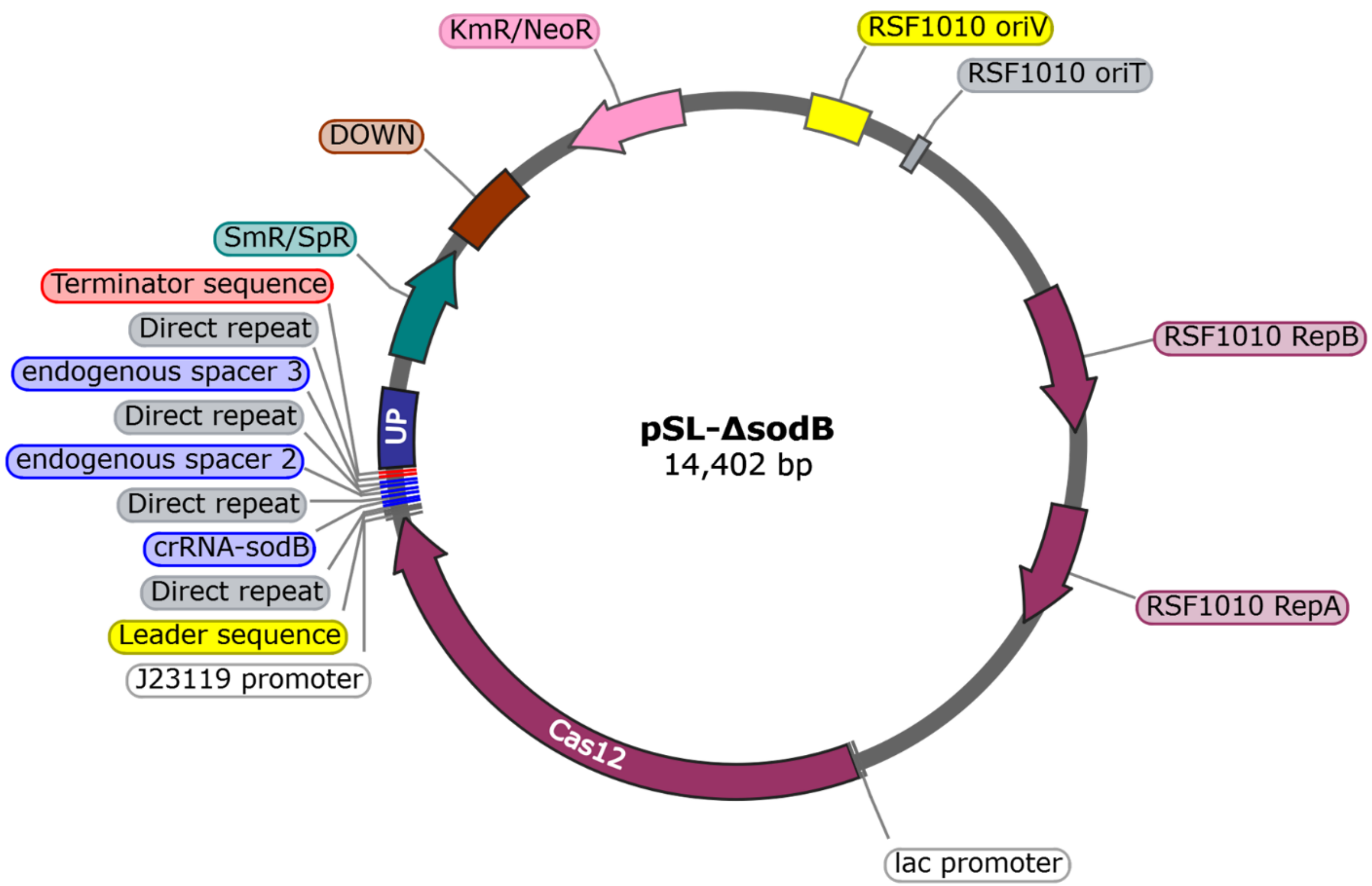

Figure S5: The map of pSL-*ΔsodB* editing vector made using SnapGene.

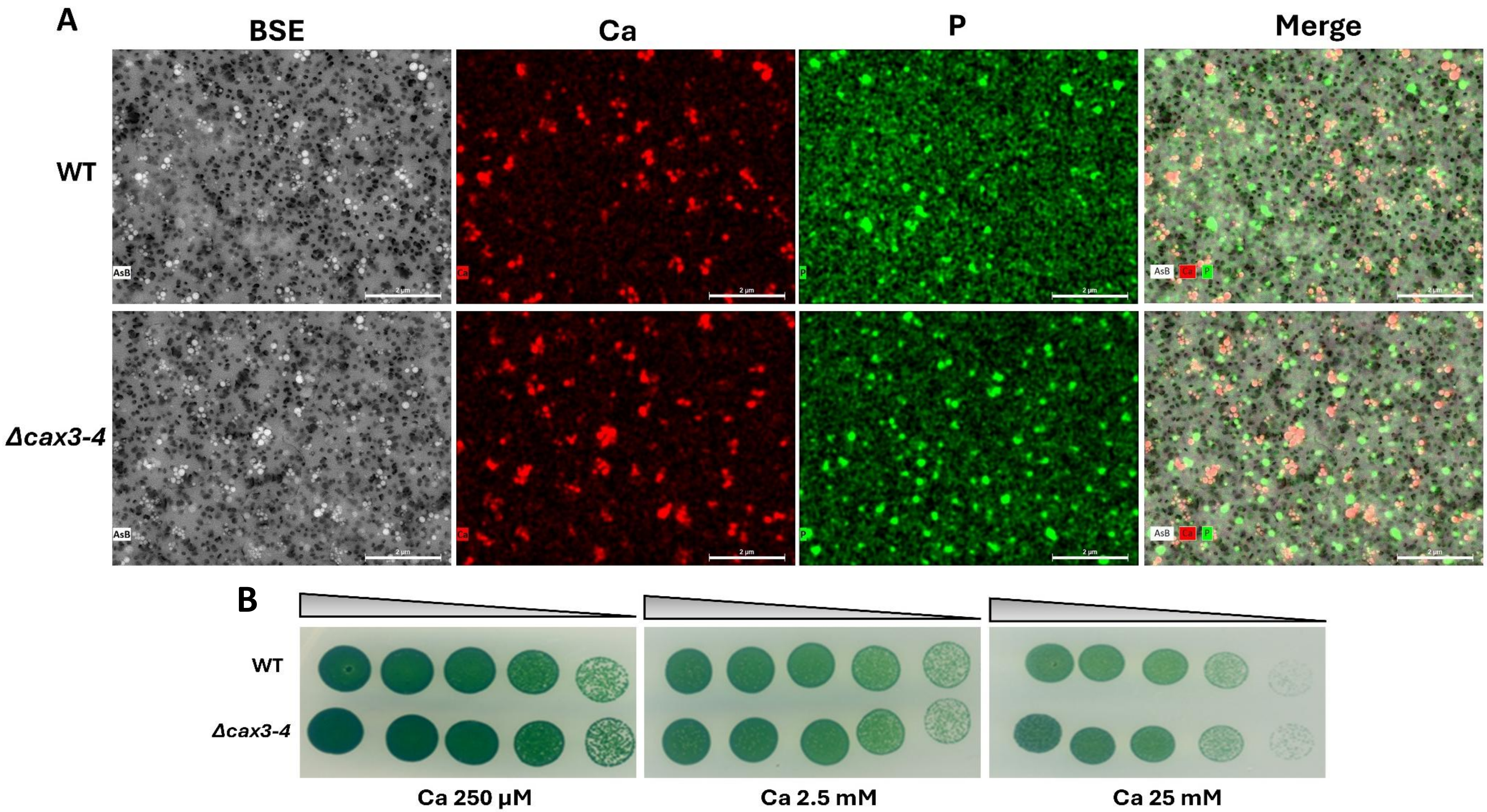

Figure S6: Phenotypic analysis of the *Δcax3-4* mutant. (A) Representative SEM images in BSE mode (leftmost panel) of WT and *Δcax3-4* cells grown in BG11 medium allowing detection of iACC inclusions (bright spots) and polyphosphate granules (light grey spots) as well as the pores (0.2 µm) of the filters (dark disks). The corresponding EDXS chemical maps (middle panels) of Ca and P are presented independently showing calcium-rich iACC inclusions as red dots and phosphate-rich PolyP granules as green dot, respectively. Merged images of the overlay of Ca and P chemical maps are shown in the rightmost panel. Scale Bar: 2 µm. Representative images from experiments performed using two independently isolated mutant strains are shown. **(B)** Spot-test assay for checking the influence of increasing calcium concentration on the growth of WT and *Δcax3-4* strains. The plates were incubated for 14 days under standard conditions prior to photography. This experiment was performed twice with independent biological replicates.

TGCCACCGACATTGCCCGAGAAACCGCCGATGTGGTGCTGATGGAAGACGATCTGCGCGGC
TTGCTGCTGGCAATCAAAGTGGCGAAACAGTCCATGGATATTATCTGGCAGAACACTATGGT
TGTCGCGGTGCCTAACATGGGAGCCCTTCTGTCCGGGATCTTCTTCGCCCTCGACCCAATTC
TGGCGGTGGTGATTAACAACGGTTCGGCGATCCTGGCTGAACTGAATGGTCTGCGTCCCCTC
ATGGGTCCAGGAGGAGTCGTTCCCCTGGGACATACTCTCACCGCTGCTGAACTGGCTGAAG
AAGACAAGCGGCTGCACGAACATCGGGAGACAACTCACCTGGTGAATGGCAAGACCGATCA
CCCGACGAATGGTAAATCCTCGATTCCAGATGTACAACTGGAGCACACGAATGGTAAATCCT
CTTCAGCTAAATCTGGGCGTGAAAAGGCTACAGCAGTTTTGGTAGGAGCCGAGAAATAGAA
ACGAAACACTCAGGGTTCTGGGGGGATTCTTCCTCAGAACTCTACCGCCTGTGTATCCCTGA
ACCTGTATACAAAAATAAAGTATGGCGCCCCTGGAACCTTTAAGTCGGAACCGTTCAGAGGC
AATCTATGCGCTCACGCAACTGGTCCAGAACCTTGACCGAACGCAGCGGTGGTAACGGCGC
AGTGGCGGTTTTCATGGCTTGTTATGACTGTTTTTTTGGGGTACAGTCTATGCCTCGGGCAT
CCAAGCAGCAAGCGCGTTACGCCGTGGGTCGATGTTTGATGTTATGGAGCAGCAACGATGT
TACGCAGCAGGGCAGTCGCCCTAAAACAAAGTTAAACATCATGAGGGAAGCGGTGATCGCC
GAAGTATCGACTCAACTATCAGAGGTAGTTGGCGTCATCGAGCGCCATCTCGAACCGACGTT
GCTGGCCGTACATTTGTACGGCTCCGCAGTGGATGGCGGCCTGAAGCCACACAGTGATATT
GATTTGCTGGTTACGGTGACCGTAAGGCTTGATGAAACAACGCGGCGAGCTTTGATCAACGA
CCTTTTGGAAACTTCGGCTTCCCCTGGAGAGAGCGAGATTCTCCGCGCTGTAGAAGTCACCA
TTGTTGTGCACGACGACATCATTCCGTGGCGTTATCCAGCTAAGCGCGAACTGCAATTTGGA
GAATGGCAGCGCAATGACATTCTTGCAGGTATCTTCGAGCCAGCCACGATCGACATTGATCT
GGCTATCTTGCTGACAAAAGCAAGAGAACATAGCGTTGCCTTGGTAGGTCCAGCGGCGGAG
GAACTCTTTGATCCGGTTCCTGAACAGGATCTATTTGAGGCGCTAAATGAAACCTTAACGCT
ATGGAACTCGCCGCCCGACTGGGCTGGCGATGAGCGAAATGTAGTGCTTACGTTGTCCCGC
ATTTGGTACAGCGCAGTAACCGGCAAAATCGCGCCGAAGGATGTCGCTGCCGACTGGGCAA
TGGAGCGCCTGCCGGCCCAGTATCAGCCCGTCATACTTGAAGCTAGACAGGCTTATCTTGGA
CAAGAAGAAGATCGCTTGGCCTCGCGCGCAGATCAGTTGGAAGAATTTGTCCACTACGTGAA
AGGCGAGATCACCAAGGTAGTCGGCAAATAATGTCTAACAATTCGTTCAAGCCGACGCCGG
ATCAAATCCGGTCAGGATGGGGAATGTAGTCACACTAGAAGGAAATAATCAAATAGGGTTCC
TCAAACTTGCCTGCCTTGACCGGCTGCCCTTTCAAATCTTCTGGCAAAGCAATATTTCGGCG
TTGATCTCCCGCTTCAACCGTCAGATCAGACCCGTACTGGGTTAGTTTGACTTGATGGCGTT
GGAAACCCGGCAGAAAAACCAGAATCTGGCGCTGCTCCCGATCGAACTGCAGGGGGGGAGG
AACCGCCGGGATCGCTTCTAAGTCGGGGAGGGCCTCCAGCAAAGGTTGCCAGTTCAGGTCC
TGCAGAGGCGGAATTCCATGCACACCTAAAGGAGCAAAATTCTGCTGCAGGTTGTCCAGCCC
AACGTCGCCCTGTGGATAGACCAGCACCCCACTGACGCCTAAATTAACCTGTTGAGCACTCC
CCCAGAGCCAACGGGATTGGGCGATCGCCGCCGCATCAGCACCAGTGACCAAATAGGCGGT
TAATTGACGGGCATCACTCACCACCGTTACCCCCTGAGCAATCCAGGTTTTCACCTGTTCTA
ATCCCTGTTGGACTTTATCACTATCGATATTGGCTGAGAGCAGGGCGCTGGCGATCGGTCCC
CCCAGGGAAG

Figure S7: Sequence of the homology repair template cloned in the editing vector pSL-*Δcax3-4.* The upstream region, $Sm^{R}Sp^{R}$ cassette and the downstream region are shown in light blue, turquoise and yellow, respectively.

**GGGATTGCCTTGCTACCAACCCACTCCGTCCAGGCCCAGGGGGTCAAATTTTTCTGTGATGA
GGCTAATGCCATTCCCACCACCACCGCCCGCACGGCCCAGGGCAACGTTGCTATTATCCGCT
GGGCTTCCACCCTGGGAGATAACTACGATCCCCTCACCCGTTGTCGTCAGGTTTCCGCCCGA
TTCCAATCCTTCTATGACCAGCGCTTGCTGCGGTTTCTCACCACGGGGCGGATGAATGGTCA
GAATGTGGTTTGTGTTGCGCTCAAGCGAGGCGAAGACTGTATCCGCCCCAACGGCTTACTCT
TTACACTCAAACCCGGAAGTAATCCCAGAAATACGCTCTTACAACTGGTGAATGTGAGTAAC
AAGGTGGGCGGGCCCCTGTATGAAAGTTCGGCGGAACGGGTTTATCTGGATATGGACCAGG
TCCTCAATCCCCCCGAAACGATCGCCAGCCCATCCCCTGAACCGGCTCCAACCTCGCCCTAA
GCCAGGATCCCGGGCAGACTTGACCTGATAGTTTGGCTGTGAGCAATTATGTGCTTAGTGCA
TCTAACGCTTGAGTTAAGCCGCGCCGCGAAGCGGCGTCGGCTTGAACGAATTGTTAGACATT
ATTTGCCGACTACCTTGGTGATCTCGCCTTTCACGTAGTGGACAAATTCTTCCAACTGATCTG
CGCGCGAGGCCAAGCGATCTTCTTCTTGTCCAAGATAAGCCTGTCTAGCTTCAAGTATGACG
GGCTGATACTGGGCCGGCAGGCGCTCCATTGCCCAGTCGGCAGCGACATCCTTCGGCGCGA
TTTTGCCGGTTACTGCGCTGTACCAAATGCGGGACAACGTAAGCACTACATTTCGCTCATCG
CCAGCCCAGTCGGGCGGCGAGTTCCATAGCGTTAAGGTTTCATTTAGCGCCTCAAATAGATC
CTGTTCAGGAACCGGATCAAAGAGTTCCTCCGCCGCTGGACCTACCAAGGCAACGCTATGTT
CTCTTGCTTTTGTCAGCAAGATAGCCAGATCAATGTCGATCGTGGCTGGCTCGAAGATACCT
GCAAGAATGTCATTGCGCTGCCATTCTCCAAATTGCAGTTCGCGCTTAGCTGGATAACGCCA
CGGAATGATGTCGTCGTGCACAACAATGGTGACTTCTACAGCGCGGAGAATCTCGCTCTCTC
CAGGGGAAGCCGAAGTTTCCAAAAGGTCGTTGATCAAAGCTCGCCGCGTTGTTTCATCAAGC
CTTACGGTCACCGTAACCAGCAAATCAATATCACTGTGTGGCTTCAGGCCGCCATCCACTGC
GGAGCCGTACAAATGTACGGCCAGCAACGTCGGTTCGAGATGGCGCTCGATGACGCCAACT
ACCTCTGATAGTTGAGTCGATACTTCGGCGATCACCGCTTCCCTCATGATGTTTAACTTTGTT
TTAGGGCGACTGCCCTGCTGCGTAACATCGTTGCTGCTCCATAACATCAAACATCGACCCAC
GGCGTAACGCGCTTGCTGCTTGGATGCCCGAGGCATAGACTGTACCCCAAAAAAACAGTCAT
AACAAGCCATGAAAACCGCCACTGCGCCGTTACCACCGCTGCGTTCGGTCAAGGTTCTGGAC
CAGTTGCGTGAGCGCATACCCGGGCGTCTTCCCTCCTCGCAGATCTGAATGGCAGTCTGGAT
CGCTTTATTTCTCTATTCTGGCCTATGACATGCGACTCGTCGATCAGGCTTGATCCTTAATGG
CCAATCCCGACGTAACGGAACCCAGCAGCTTCCATCATTTCCCGACCCAGGAAATTACGCCC
ATCCACCAGGACAGGGTTGGTCATCAGCTGAGCCATCTTGGTGTAATCCAGGGTCTTGAACT
GTTGCCATTCCGTCACCAGCACCAGGGCATCACAACCATCCGCCAGCCGTTCCGGGTCCGTT
TCCACCAGCACGTTAGACAGGCCATGCCGCATTCCGGTTTGGGAGACGAGGGGATCGTAGG
CTTTTACCTTGGTGCCCAGCCGGGTCAATTGCTCAATCAGGCTGAGGGCAGGGGCATCGCG
CATATCGTCGGTGTTGGGTTTGAAGGTTAGGCCCAGCAGTCCCACGGTTTTGCCCTTAAGGA
TTTTCAGCATTTGCTGCAGTTTTTCGATTACCACCTGACGTTGCCGTT**

Figure S8: Sequence of the homology repair template cloned in the editing vector pSL-*Δgor*. The upstream region, $Sm^R Sp^R$ cassette and the downstream region are shown in orange, turquoise and green, respectively.

TCTCCGGTATGCCTGCCCTCCCCCCCGTTGCAGCTTCATCCCCAAACCGTGCTTGCTAACTC
CTTTGGTGGTTTCAGTCCAGCGGAGCAACCAGAAAGCTTAACCCGATCGGCAGAGATGGTC
AGCTCAGTGTCGATCGCTCCCGCCGAAGATGAATGGTTCAAACAAGCCGATCAGGTGCTCTG
GCAAGCCCGCTGTCTGGCCAATGAATGGCGCGGTTCCCTCAAAACCCATCTGCGCTTCGGCC
AGTTGGGGGAACAGTTAGAGCAGGTGGTAAGCATGGAGCGAGCGAGCCTGTTAATTTTGGG
TTGTCTGGGTACAGAACATCCTCTGGTGCAACAATTGAGCCGAGATTTGCCCTGTCCCGTCT
TGGGAATCCCCCTGGCTGCAATTGGTTAAGGAGAAGAGAATTCCCTAGAATGTTTAATGGCA
TCGCTACGGCAGGTGGAGTAGCCTCATCCACTTCATCTCTACCTCTGCCGTCTAGGCAATGC
TGAAGCAGTTAAACAAGGAGAACACCTATGCGCTCACGCAACTGGTCCAGAACCTTGACCGA
ACGCAGCGGTGGTAACGGCGCAGTGGCGGTTTTCATGGCTTGTTATGACTGTTTTTTTGGGG
TACAGTCTATGCCTCGGGCATCCAAGCAGCAAGCGCGTTACGCCGTGGGTCGATGTTTGATG
TTATGGAGCAGCAACGATGTTACGCAGCAGGGCAGTCGCCCTAAAACAAAGTTAAACATCAT
GAGGGAAGCGGTGATCGCCGAAGTATCGACTCAACTATCAGAGGTAGTTGGCGTCATCGAG
CGCCATCTCGAACCGACGTTGCTGGCCGTACATTTGTACGGCTCCGCAGTGGATGGCGGCC
TGAAGCCACACAGTGATATTGATTTGCTGGTTACGGTGACCGTAAGGCTTGATGAAACAACG
CGGCGAGCTTTGATCAACGACCTTTTGGAAACTTCGGCTTCCCCTGGAGAGAGCGAGATTCT
CCGCGCTGTAGAAGTCACCATTGTTGTGCACGACGACATCATTCCGTGGCGTTATCCAGCTA
AGCGCGAACTGCAATTTGGAGAATGGCAGCGCAATGACATTCTTGCAGGTATCTTCGAGCCA
GCCACGATCGACATTGATCTGGCTATCTTGCTGACAAAAGCAAGAGAACATAGCGTTGCCTT
GGTAGGTCCAGCGGCGGAGGAACTCTTTGATCCGGTTCCTGAACAGGATCTATTTGAGGCG
CTAAATGAAACCTTAACGCTATGGAACTCGCCGCCCGACTGGGCTGGCGATGAGCGAAATG
TAGTGCTTACGTTGTCCCGCATTTGGTACAGCGCAGTAACCGGCAAAATCGCGCCGAAGGAT
GTCGCTGCCGACTGGGCAATGGAGCGCCTGCCGGCCCAGTATCAGCCCGTCATACTTGAAG
CTAGACAGGCTTATCTTGGACAAGAAGAAGATCGCTTGGCCTCGCGCGCAGATCAGTTGGA
AGAATTTGTCCACTACGTGAAAGGCGAGATCACCAAGGTAGTCGGCAAATAATGTCTAACAA
TTCGTTCAAGCCGACGCCGCTTCGCGGCGCGGCTTAACTCAAGCGTTAGATGCACTAAGCAC
ATAATTGCTCACAGCCAAACTATCAGGTCAAGTCTGGTCTGCTAGGCGTTACTACATCTCCT
GGTAGACCAAAGATGGCCTGAATTCAGCCGCATACGCCATTTCAACTGATTTGCAATTCGCT
TAACCCTGCCCCAGCGGGGTTTTTATCGTTTTATGGCCCAGTTGGTCCCAGCACAGCGATCG
CGGCTAGCTAAGGATTGGTGACTGGCTGACCCGGTTCCCCGGCAGGAATGAACGCAGCCCG
ATCAATTGGCCAGAAGCGAATCGAAGCCCGCCCAATAATGTTTTTCTGTGGCACAAATCCCC
AGCAGCGGCCATCAAAACTGTTGTCCCGATTATCTCCTAACACTAAATAAGCCTGGGGAGGA
ACTTTCTGGGGCTGGGACAGAAAAGCTTGGCCATTACAGGTGTCGATCGTGGTTTCAACCCC
AGAGGGGAGATAATTTTCCTGCAGGGGACGTCCATTGATGTAAACTTTACCGTTCTCGATCG
CCACCTCATCCCCCGGTAAACCAACGATGCGCTTGATAAAAGCATCTTTAAACCCATCCTT

Figure S9: Sequence of the homology repair template cloned in the editing vector pSL-*ΔsodB*. The upstream region, $Sm^R Sp^R$ cassette and the downstream region are shown in purple, turquoise and brown, respectively.

Table S1: List of plasmids used in this study.

Plasmid	Features	Reference
pC	RSF1010 derivative harboring the λ phage promoter allowing strong constitutive expression. Used as donor of Sm^R/Sp^R cassette	(Chenebault et al., 2020)
pSB2T	RSF1010 derivative harboring tac promoter. Used as control for conjugation experiments.	(Marraccini et al., 1993)
pSL2680	RSF1010 derivative harboring the *cas12a* gene that is expressed from a lac promoter and the endogenous CRISPR array from *Francisella novicida* from a J23119 promoter.	(Ungerer and Pakrasi, 2016)
pSL2680-trCas12a	pSL2680 vector with truncated Cas12a (removal of amino acids 733-1330) that lacks the RuvC and nuclease domain	This study
pSL-*Δcax3-4*	pSL2680 vector with crRNA targeting *cax3* and Sm^R/Sp^R cassette flanking homology platforms (~600 bp) for deletion of *cax3-4* genes	This study
pT-*Δgor*	pTwist plasmid with Sm^R/Sp^R cassette flanking homology platforms for *gor* deletion	This study
pSL-*Δgor*	pSL2680 vector with crRNA targeting *gor* and Sm^R/Sp^R cassette flanking homology platforms (~500 bp) for deletion of *gor* gene	This study
pSL-*ΔsodB*	pSL2680 vector with crRNA targeting *sodB* and Sm^R/Sp^R cassette flanking homology platforms (~500 bp) for deletion of *sodB* gene	This study

Table S2: List of primers used in this study.

Primer Name	Sequence	Feature
gRNAseq-1	TCTTGACAGCTAGCTCAGTCC	Verification and sequencing of pSL2680 plasmids
gRNAseq-2	GACCGGCTACTAATACAAAAGG	
cas12_Fw	TTAGAATAAGAAATCATTCCACACA	Amplification of internal fragment of the *cas12* gene on pSL2680 plasmid
cas12_Rv	CTTAGACCCGTTTTTGCCTA	
SmSp-1	GATTTGCTGGTTACGGTGAC	Amplification of internal fragment of the Sm^R/Sp^R cassette
SmSp-2	CCGACTACCTTGGTGATCTC	
**Construction of pSL-crRNA- Δcax3-4**		
cr-cax3-4_Fw	agatATTGGTTCCATTATTGCCAACC	Construction of crRNA targeting *cax3* gene
cr-cax3-4_Rv	agacGGTTGGCAATAATGGAACCAAT	
F1-cax_Fw	AGCTTTAATGCGGTAGTTGGTACCGT CGACTGCCACCGACATTGCCCGA	~600 bp upstream region from ATG of *cax3* gene
F1-cax_Rv	GCGTGAGCGCATAGATTGCCTCTGA ACGGTTCCG	
F2-cax_Fw	TCAGAGGCAATCTATGCGCTCACGC AACTGG	Amplification of Sm^R/Sp^R cassette from pC
F2-cax_Rv	CCGGATTTGATCCGGCGTCGGCTTG AACG	
F3-cax_Fw	CAAGCCGACGCCGGATCAAATCCGG TCAGGATGGGGAAT	~600 bp downstream region from stop codon of *cax4* gene
F3-cax_Rv	TGCCCGGATTACAGATCCTCTAGAG TCGACCTTCCCTGGGGGGACCGA	
**Verification of *Δcax3-4***		
cax-1	ATTAATGACTCTGCCGCCCT	Amplification outside the upstream and downstream region of *cax3-cax4* gene
cax-2	ATCTGGGCACGTTACGGTTA	
cax-3	TTCTGTTGGGGATGCTAGCC	Amplification of internal fragment of the *cax4* gene
cax-4	CAGGGCAATTTGGAGACTGG	
**Construction of pSL-crRNA- Δgor**		
cr-gor_Fw	agatCAGGGATCATGCATGGGTTGGG	Construction of crRNA targeting *gor* gene
cr-gor_Rv	agacCCCAACCCATGCATGATCCCTG	
F-pT-Δgor_Fw	AGCTTTAATGCGGTAGTTGGTACCGT CGAC CCAGTCACGACGTTGTAAAAC	Cloning of homology repair template from pT-Δgor to pSL- crRNA-Δgor
F-pT- Δgor_Rv	TGCCCGGATTACAGATCCTCTAGAG TCGAC TTCACACAGGAAACAGCTATGACCA T	
**Verification of *Δgor***		
gor-1	ACAACTGGGTGTACCCTTGC	Amplification outside the upstream and downstream region of *gor* gene
gor-2	ACAACTGGGTGTACCCTTGC	
gor-3	TTCAGAAAGACCGACGGTAG	

gor-4	GTCCTATGGAGCAAAAGTGG	Amplification of internal fragment of the *gor* gene
**Construction of pSL-crRNA- ΔsodB**		
cr-sodB_Fw	agatAGTTTCACTATGGCAAGCACCA	Construction of crRNA targeting *sodB* gene
cr-sodB_Rv	agacTGGTGCTTGCCATAGTGAAACT	
F1-sodB_Fw	CGGTAGTTGGTACCGTCTCCGGTATGCCTGCCCT	~500 bp upstream region from ATG of *sodB* gene
F1-sodB_Rv	AGCGCATAGGTGTTCTCCTTGTTTAACTGCT	
F2-sodB_Fw	AGAACACCTATGCGCTCACGCAACTGG	Amplification of Sm^R/Sp^R cassette from pC
F2-sodB_Rv	CTAGCAGACCAGACTTGACCTGATAGTTTGGCTGTG	
F3-sodB_Fw	TCAAGTCTGGTCTGCTAGGCGTTACTACATCTCC	~500 bp dowstream region from stop codon of *sodB* gene
F3-sodB_Rv	CGGATTACAGATCCTCTAGAGAAGGATGGGTTTAAAGATGCTTTTATCAAGC	
**Verification of *ΔsodB***		
sod-1	CCGTAATTGTGCAGGTCGTC	Amplification outside the upstream and downstream region of *sodB* gene
sod-2	CTGCAAATCAACGATCGCCT	
sod-3	GCCTCCCTTACCCTACGATC	Amplification of internal fragment of the *sodB* gene
sod-4	GGCAACAAAGTCCCAGTTCA	

UNCROPPED GEL IMAGES

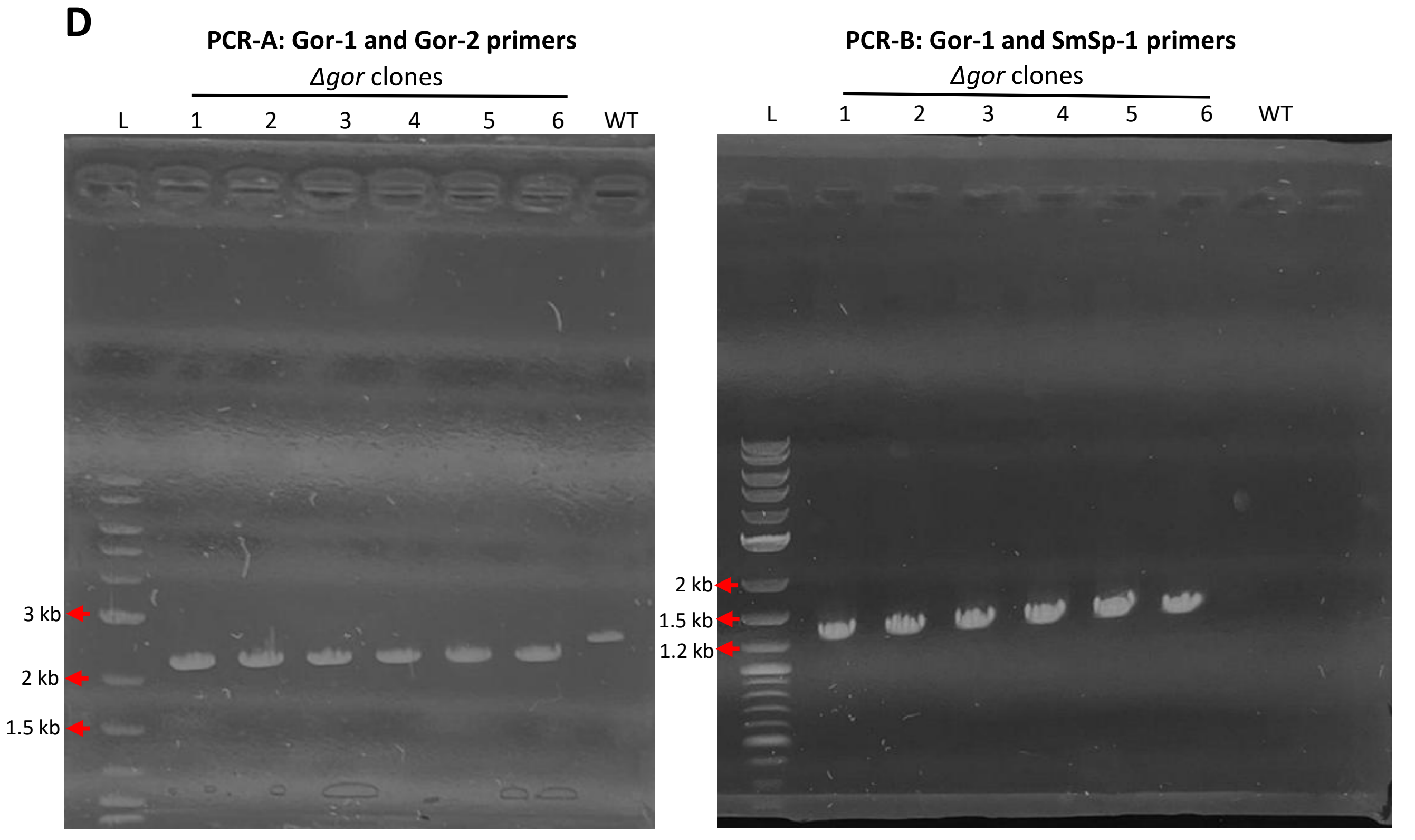

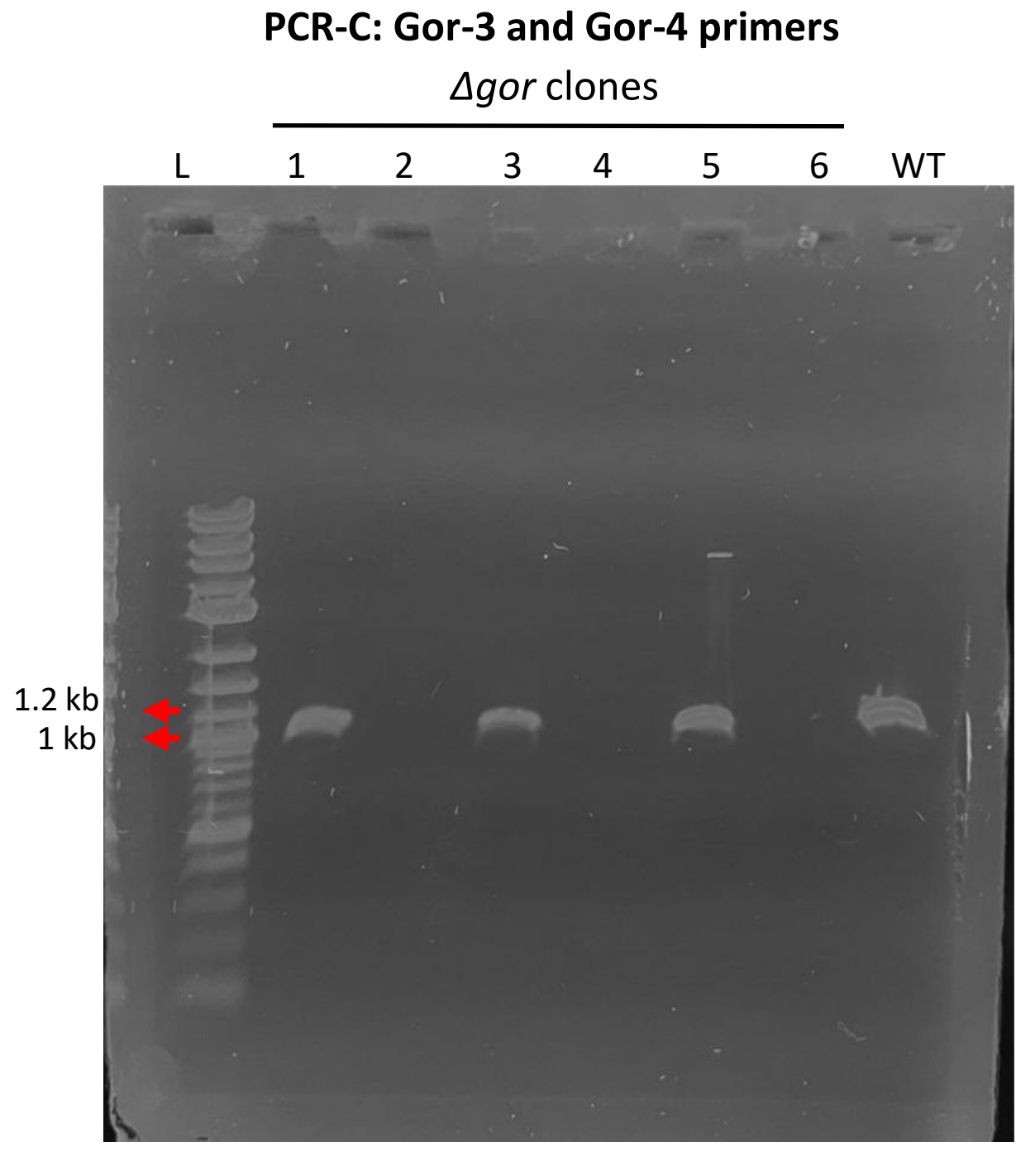

Expected size of PCR products

Primers / Strains	PCR-A: Gor-1 and Gor-2	PCR-B: Gor-1 and SmSp-1	PCR-C: Gor-3 and Gor-4
**WT**	2480 bp	-	1024 bp
Δgor	2236 bp	1301 bp	-

Figure 2: Targeted deletion of the *gor* gene in *Cyanothece* PCC 7425. (D) Three PCR strategies were employed to confirm successful deletion of the *gor* gene in six Sm^RSp^R clones. PCR products (expected sizes indicated in table) were resolved on 1% agarose gels. L denotes DNA ladder (1 kb plus, NEB).

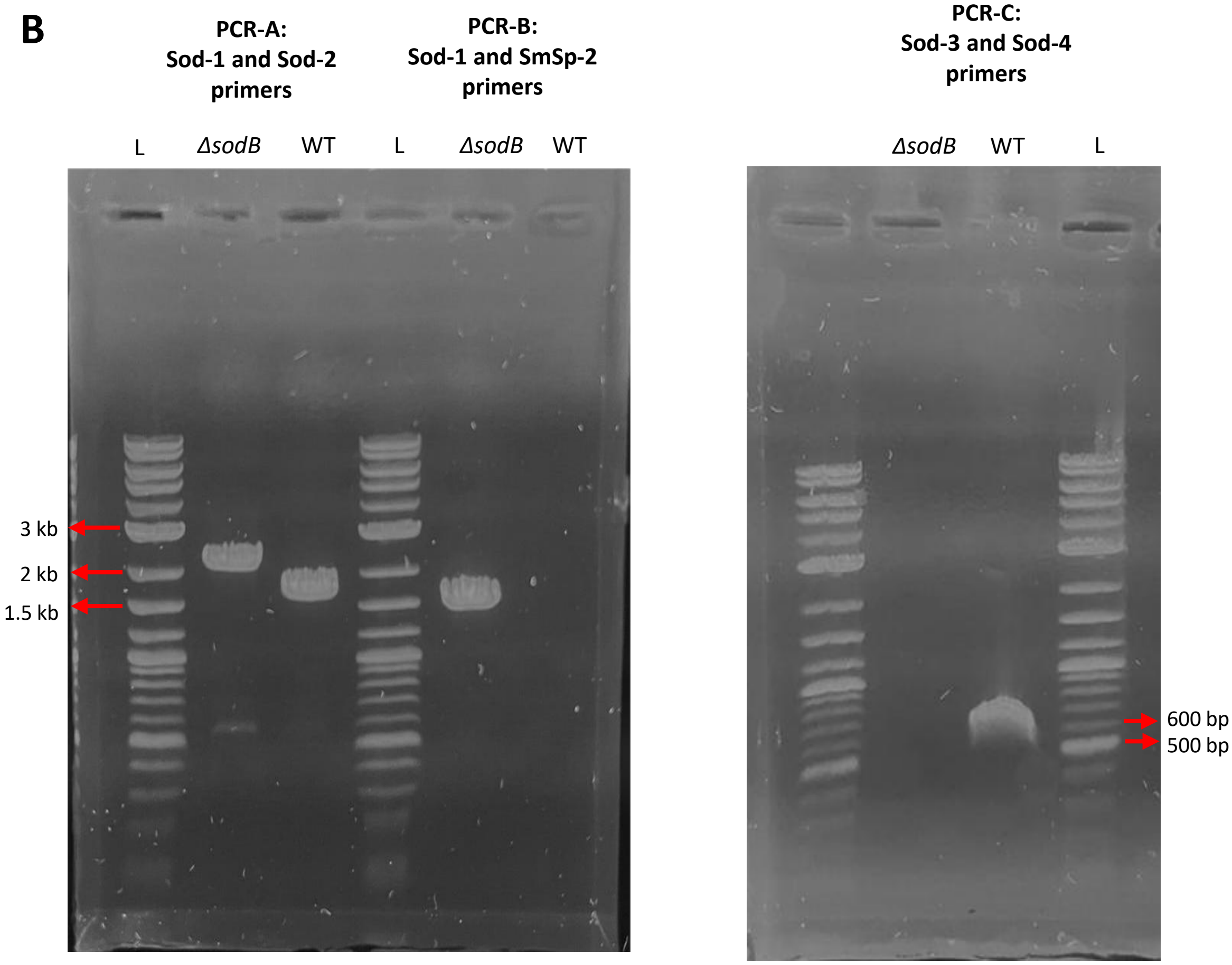

Expected size of PCR products

Primers / Strains	PCR-A: Sod-1 and Sod-2	PCR-B: Sod-1 and SmSp-2	PCR-C: Sod-3 and Sod-4
**WT**	1852 bp	-	568 bp
ΔsodB	2372 bp	1635 bp	-

Figure 3: Targeted deletion of the *sodB* gene in *Cyanothece* PCC 7425. (B) Three PCR strategies were employed to confirm successful deletion of the *sodB* gene. PCR products (expected sizes indicated in table) were resolved on 1% agarose gels. L denotes DNA ladder (1 kb plus, NEB).

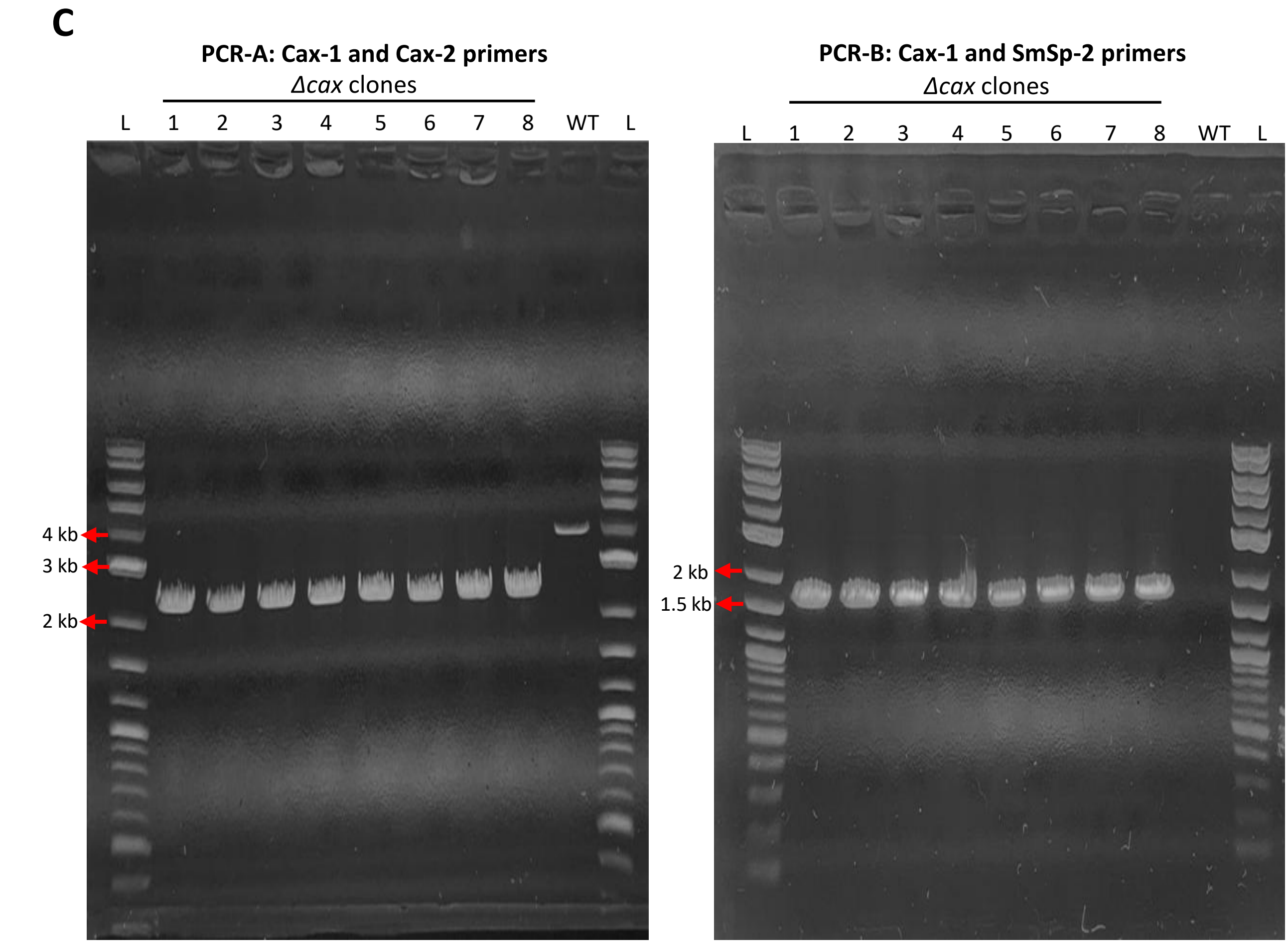

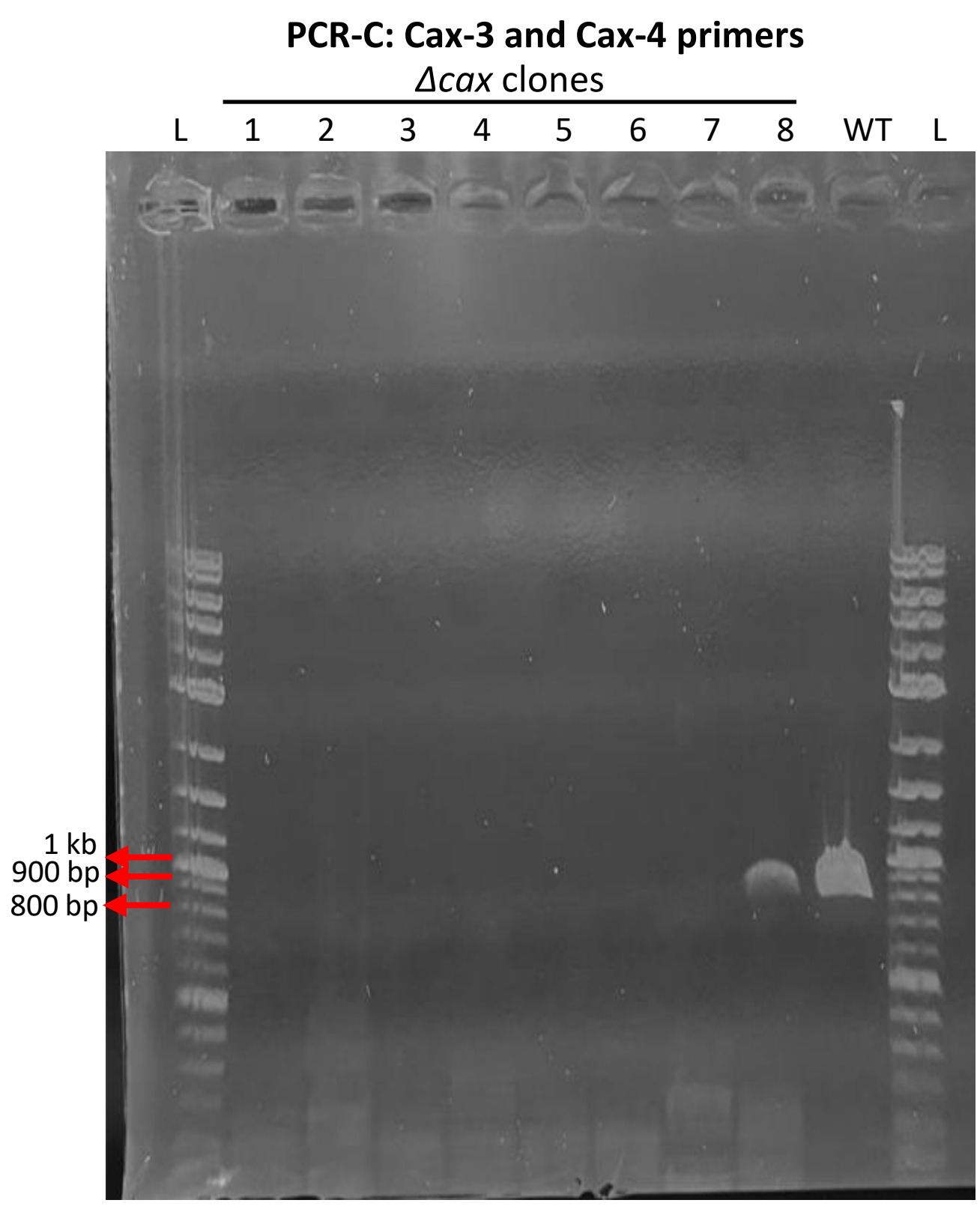

Expected size of PCR products

Strains \ Primers	PCR-A: Cax-1 and Cax-2	PCR-B: Cax-1 and SmSp-2	PCR-C: Cax-3 and Cax-4
**WT**	3955 bp	-	851 bp
Δcax	2419 bp	1665 bp	-

Figure 4: Targeted deletion of *cax3* and *cax4* genes in *Cyanothece* PCC 7425. (C) Three PCR strategies were employed to confirm successful deletion of the *cax3* and *cax4* genes in eight Sm^RSp^R clones, showing complete absence of WT chromosome copy in the *Δcax3-4* clone n° 1-7. PCR products (sizes indicated in table) were resolved on 1% agarose gels. L denotes DNA ladder (1 kb plus, NEB).

D

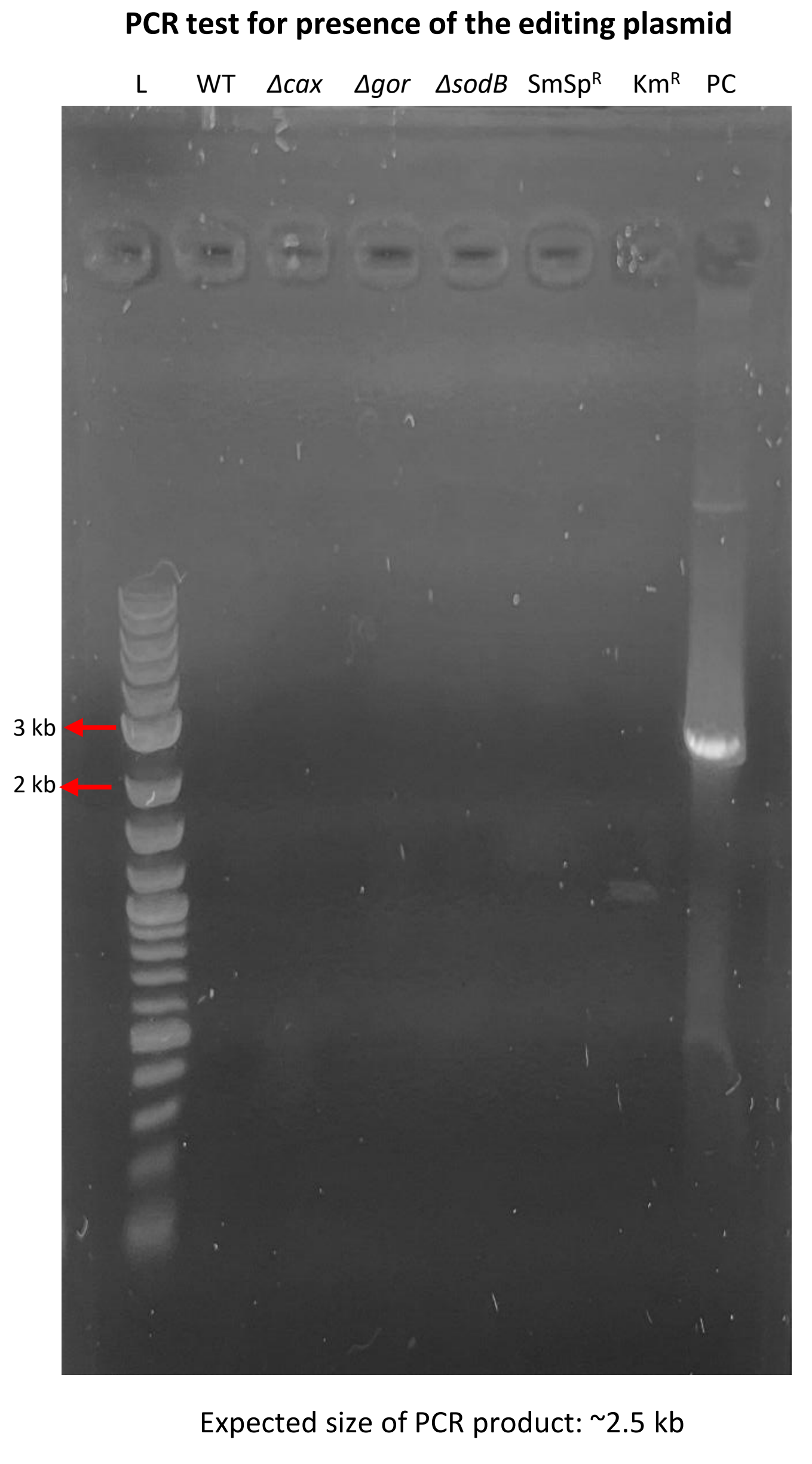

Figure 5: Verification of presence of the editing plasmid. (D) PCR analysis using primers (Table S2) targeting the editing vector showing the products resolved on 1% agarose gels. PC is positive control. L denotes DNA ladder (1 kb plus, NEB)
